# From Proteome to Potential Drugs: Integration of Subtractive Proteomics and Ensemble Docking for Drug Repurposing against *Pseudomonas aeruginosa* RND Superfamily Proteins

**DOI:** 10.3390/ijms25158027

**Published:** 2024-07-23

**Authors:** Gabriela Urra, Elizabeth Valdés-Muñoz, Reynier Suardiaz, Erix W. Hernández-Rodríguez, Jonathan M. Palma, Sofía E. Ríos-Rozas, Camila A. Flores-Morales, Melissa Alegría-Arcos, Osvaldo Yáñez, Luis Morales-Quintana, Vívian D’Afonseca, Daniel Bustos

**Affiliations:** 1 Laboratorio de Bioinformática y Química Computacional, Departamento de Medicina Traslacional, Facultad de Medicina, Universidad Católica del Maule, Talca 3480094, Chile; gabriela.urra@alu.ucm.cl (G.U.); ehernandez@ucm.cl (E.W.H.-R.); sofia.rios@alu.ucm.cl (S.E.R.-R.); 2 Doctorado en Biotecnología Traslacional, Facultad de Ciencias Agrarias y Forestales, Universidad Católica del Maule, Talca 3480094, Chile; elizabeth.valdes@alu.ucm.cl; 3 Departamento de Química Física, Facultad de Ciencias Químicas, Universidad Complutense de Madrid, 28040 Madrid, Spain; reysuard@ucm.es; 4 Unidad de Bioinformática Clínica, Centro Oncológico, Facultad de Medicina, Universidad Católica del Maule, Talca 3480094, Chile; 5 Facultad de Ingeniería, Universidad de Talca, Curicó 3344158, Chile; jonathan.palma@utalca.cl; 6 Magíster en Ciencias de la Computación, Universidad Católica del Maule, Talca 3460000, Chile; camila.flores.01@alu.ucm.cl; 7 Núcleo de Investigación en Data Science, Facultad de Ingeniería y Negocios, Universidad de las Américas, Santiago 7500000, Chile; malegriaa@udla.cl (M.A.-A.); oyanez@udla.cl (O.Y.); 8 Multidisciplinary Agroindustry Research Laboratory, Instituto de Ciencias Biomédicas, Facultad de Ciencias de la Salud, Universidad Autónoma de Chile, Cinco Pte. N° 1670, Talca 3467987, Chile; luis.morales@uautonoma.cl; 9 Departamento de Ciencias Preclínicas, Facultad de Medicina, Universidad Católica del Maule, Ave. San Miguel 3605, Talca 3466706, Chile

**Keywords:** antimicrobial resistance, efflux pump, ensemble-docking, drug repurposing, *Pseudomonas aeruginosa*, subtractive proteomics, RND superfamily

## Abstract

*Pseudomonas aeruginosa* (*P. aeruginosa*) poses a significant threat as a nosocomial pathogen due to its robust resistance mechanisms and virulence factors. This study integrates subtractive proteomics and ensemble docking to identify and characterize essential proteins in *P. aeruginosa*, aiming to discover therapeutic targets and repurpose commercial existing drugs. Using subtractive proteomics, we refined the dataset to discard redundant proteins and minimize potential cross-interactions with human proteins and the microbiome proteins. We identified 12 key proteins, including a histidine kinase and members of the RND efflux pump family, known for their roles in antibiotic resistance, virulence, and antigenicity. Predictive modeling of the three-dimensional structures of these RND proteins and subsequent molecular ensemble-docking simulations led to the identification of MK-3207, R-428, and Suramin as promising inhibitor candidates. These compounds demonstrated high binding affinities and effective inhibition across multiple metrics. Further refinement using non-covalent interaction index methods provided deeper insights into the electronic effects in protein–ligand interactions, with Suramin exhibiting superior binding energies, suggesting its broad-spectrum inhibitory potential. Our findings confirm the critical role of RND efflux pumps in antibiotic resistance and suggest that MK-3207, R-428, and Suramin could be effectively repurposed to target these proteins. This approach highlights the potential of drug repurposing as a viable strategy to combat *P. aeruginosa* infections.

## 1. Introduction

Each year, around 2 million individuals in the United States suffer infections caused by antibiotic-resistant bacteria, leading to approximately 23,000 deaths due to multi-drug resistant (MDR) infections. At the same time, Europe contends with a similar scenario of roughly 25,000 deaths annually [1]. The economic impact of these infections surpasses 2 billion dollars annually, affecting the healthcare system and citizen’s finances [2]. In 2024, the World Health Organization (WHO) designated seven bacteria as high-priority pathogens due to their capacity to induce deadly contagions. Among these are *Salmonella Typhi*, *Shigella* spp., *non-typhoidal Salmonella*, and *Neisseria gonorrhoeae*, all of them resistant to fluoroquinolone. *Enterococcus faecium* vancomycin-resistant, *Staphylococcus aureus* methicillin-resistant, and *Pseudomonas aeruginosa* carbapenem-resistant [3].

*P. aeruginosa* is an opportunistic gram-negative bacterium with MDR and is the cause of acute and chronic infections in immunocompromised individuals suffering from certain pathologies such as cancer, cystic fibrosis, trauma, chronic obstructive pulmonary disease, sepsis, burns, ventilator-associated pneumonia, and is also the main cause of nosocomial disease [4,5,6]. Concerning healthcare-associated *P. aeruginosa* infections, it is estimated that account for 16.2% of patient infections and contribute to 23% of all infections acquired in the Intensive Care Unit (ICU) [7].

This bacterium can adapt to diverse and adverse host environments through the secretion of virulence factors; moreover, these virulence factors are involved in infection, colonization, and antibiotic resistance [8]. Among the virulence factors, a finite number of polar pili expressed by *P. aeruginosa* are involved in host colonization. It also presents polar flagella which determines the motility of the bacterium [9,10], both flagella and pili are responsible for antibiotic resistance due to the formation of biofilms [11]. On the other hand, the resistance-nodulation-division (RND) efflux pumps play an important role in obtaining and expressing the phenotype that gives MDR to *P. aeruginosa* [12]. The function of these pumps is based on a tripartite complex formed by the RND protein, the membrane fusion protein (MFP) and the outer membrane protein (OMP), forming a channel through the cell envelope that transports toxic compounds out of the cell [13].

The early stage of drug discovery plays a pivotal role in identifying potential therapeutic targets and compounds, laying the groundwork for the development of effective treatments. Within the context of combating antibiotic resistance, this phase gains significance as it facilitates the identification of novel strategies to counteract the evolving mechanisms of resistance in bacteria such as *P. aeruginosa*. Two synergistic computational techniques, subtractive proteomics [14] and drug repurposing [15], emerge as promising approaches in this endeavor. Subtractive proteomics enables the systematic identification and prioritization of essential proteins within the microorganism proteome that are crucial for its survival and pathogenicity. By subtracting non-essential proteins, this technique streamlines the identification of potential drug targets, focusing efforts on those most likely to yield therapeutic benefits. Concurrently, drug repurposing employs existing pharmaceutical compounds for new therapeutic purposes. This strategy capitalizes on the knowledge of approved drugs, expediting the drug discovery process and potentially avoiding the lengthy and costly traditional drug development pipeline. By repurposing drugs to target specific proteins implicated in antibiotic resistance, this approach offers a pragmatic solution to address the urgent need for novel antimicrobial agents.

## 2. Results and Discussion

**Figure 1:**
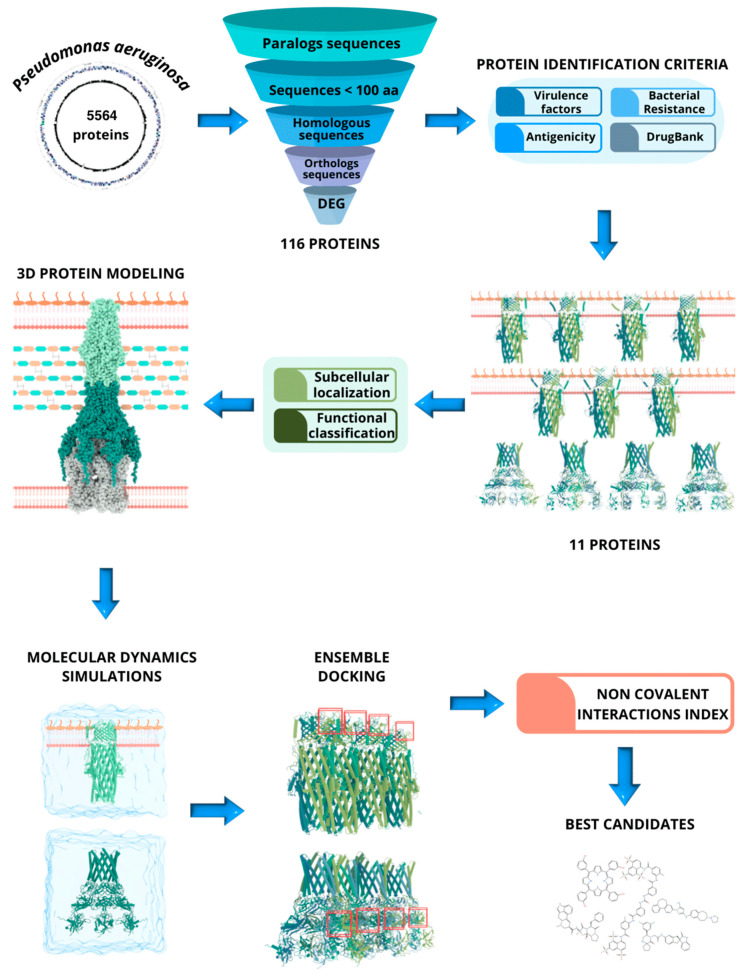
Schematic workflow: outlines each step involved in our computational methodology to first identify pharmacologically relevant proteins in *P. aeruginosa*, and subsequently predict potential drugs capable of modulating proteins in the RND family efflux pumps. The workflow includes stages of subtractive proteomics, identification of essential proteins, 3D modeling, and drug repurposing predictions through ensemble-docking and non-covalent interactions index.

The study describes a pipeline that integrates various computational techniques to identify essential and druggable proteins within the *P. aeruginosa* proteome (PAO1—ATCC 15692). Specifically, these targets belonging to the RND efflux pump were three-dimensionally modeled and energy equilibrated through Molecular Dynamics (MD) simulations for repositioning 5709 approved drugs, using ensemble docking and promolecular densities to rank their protein-drug interactions. Every stage of the sequential protocol is depicted in Figure 1.

### 2.1. Subtractive Analysis from the P. aeruginosa Proteome

The whole proteome of *P. aeruginosa* was downloaded from the NCBI database, comprising 5564 protein sequences (Table 1). To streamline the dataset and ensure the inclusion of only one copy of highly similar proteins, 111 redundant sequences were eliminated. While the significance of small proteins (sequences with a length of up to 100 amino acids) remains a debated topic due to their potential roles in various cellular processes [16], we opted to remove them. The decision was made because the functionality of most small proteins remains largely unknown, mainly due to state of art of current experimental techniques and bioinformatics tools [17]. As a result, 333 small proteins with fewer than 100 amino acids were removed from the dataset.

### 2.2. Subtractive Analysis Removing Human and Human Microbiome Orthologs and Essentiality Analysis

In our pursuit of identifying pharmacologically relevant targets with high affinity for approved drugs, it is crucial to eliminate bacterial targets with significant homology to human proteins. This step is essential for mitigating the risk of unintended side effects resulting from the modulation of these human proteins by drugs intended for bacterial targets. By employing stringent criteria to identify and exclude homologous sequences between the proteomes of *P. aeruginosa* and humans, we ensure the specificity and safety of subsequent analyses. The remaining 4344 sequences were individually compared to the 79,684 proteins belonging to the human proteome by using BLAST+. There exist 776 host proteins nearly related to the pathogen, which were removed from the subsequent analysis (Table 1).

To discover novel protein inhibitors for *P. aeruginosa*, it is imperative to underscore the pivotal role of the human microbiome, as evidenced by recent scientific investigations [18,19,20,21]. These studies elucidate the intricate bidirectional interactions between the gut microbiota and non-antibiotic drugs, revealing that the microbiome not only influences the pharmacokinetics and pharmacodynamics of drugs but is also modulated by them. Furthermore, the microbiome’s capacity to enzymatically transform drug structures underscores its potential to influence drug bioavailability, bioactivity, and toxicity, thereby shaping individual responses to pharmacotherapy. In this sense, we compare the sequences of 299,611 proteins belonging to 73 bacteria from the gut microbiome to remove 984 of them with high homology to the *P. aeruginosa* proteome (Table 1). This ensures that any potential *P. aeruginosa* inhibitors identified in our pipeline do not induce inadequate pharmacokinetic conditions, such as poor absorption, distribution, metabolization, or excretion of the drugs, while also addressing pharmacodynamic concerns, including high toxicity in humans, to safeguard patient health.

The last step of this first filter involves comparing our dataset against the DEG database. DEG contains the essential genes for more than 66 bacteria, as well as other domains of life (Archaea and Eukaryotes). Essential genes represent fundamental components vital for the survival and sustenance of an organism, thus constituting a cornerstone of its existence. In bacteria, essential genes represent the minimal yet indispensable components of the genome, forming functional modules pivotal for key biological functions. Consequently, these genes and their protein products serve as prime candidates when seeking relevant pharmacological targets. Among the dataset comprising 3360 proteins obtained previously, 96.55% of these proteins have been categorized as non-essential, while only 116 proteins fulfill the critical subcellular requirements necessary for the survival of *P. aeruginosa* (Table 1).

### 2.3. Assessing the Attributes of Virulence, Antigenicity, Bacterial Resistance, and Druggability

In the *P. aeruginosa* pathogenicity context, a multitude of virulence factors (such as proteases, lipopolysaccharides, and outer membrane proteins among others) have been documented [22] to contribute to various pathogenic mechanisms underlying nosocomial infections in immunocompromised individuals. These factors intricately orchestrate diverse strategies by which the bacterium inflicts harm upon the host’s defenses. Another relevant characteristic of the pathogenicity of *P. aeruginosa* is that this bacterium exhibits a formidable array of intrinsic and acquired resistance mechanisms, contributing to its ability to withstand classical antibiotic treatments. This underscores the urgent imperative to explore novel strategies for combating these pathogenic infections [23]. Furthermore, the identification of antigenic-related proteins holds significant importance in the exploration of novel therapeutic avenues for treating infections caused by *P. aeruginosa*. In the past, several *P. aeruginosa* antigens have been a matter of vaccine and immunotherapy development [24].

**Figure 2:**
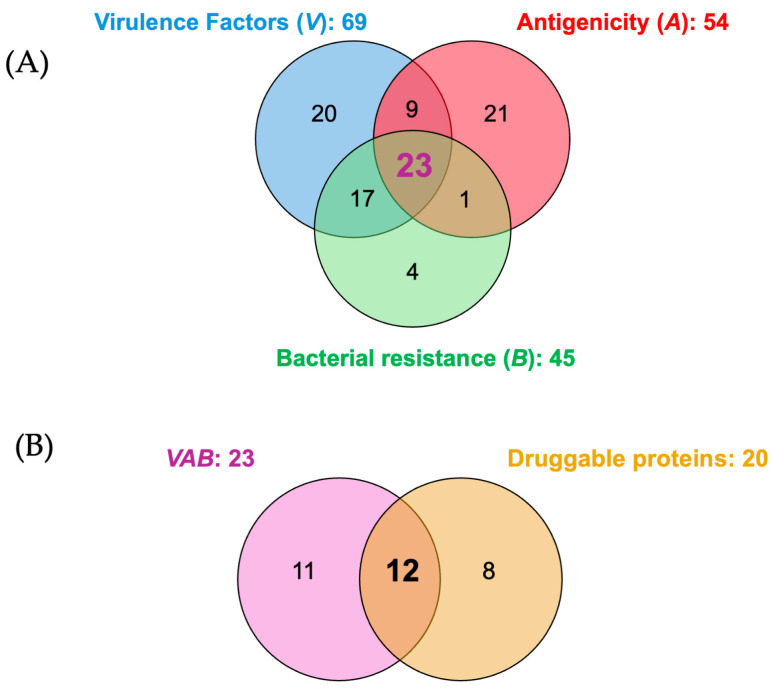
Venn diagrams depicting characteristics of *P. aeruginosa* proteins. (**A**) Evaluation of 116 most relevant proteins based on their virulence (*V*), antigenicity (*A*), and bacterial resistance (*B*) properties. (**B**) Druggability assessment of the 23 proteins that satisfy the combined criteria of virulence, antigenicity, and bacterial resistance (*VAB*).

The evaluation of the 116 previously identified proteins against the specified criteria revealed intriguing findings. Among them, 69 proteins demonstrated characteristics indicative of virulence factors, highlighting their potential role in the pathogenicity of *P. aeruginosa*. Additionally, 45 proteins exhibited features associated with conferring resistance, underscoring their contribution to the bacterium’s ability to withstand antibiotic treatments. Furthermore, 54 proteins displayed antigenic properties, suggesting their potential as targets for immune recognition and intervention. However, the intersection of these criteria revealed a select group of proteins that possess a particularly noteworthy profile. Remarkably, only 23 proteins met all three criteria simultaneously (Figure 2).

This subset of 23 proteins emerges as a compelling avenue for in-depth investigation and therapeutic targeting. Upon examination of their druggability within the DrugBank database, it was revealed that only 12 of these proteins are classified as pharmacological targets. Remarkably, each of these 12 proteins has either an FDA-approved drug or a candidate undergoing investigational phases in the drug development pipeline.

### 2.4. Characterization of Essential Proteins of P. aeruginosa

One of the proteins identified from the previous selection is a histidine kinase (HK; Table 2), which plays a critical role in coordinating resistance mechanisms and pathogenicity through its role in two-component systems (TCSs). These systems allow the bacterium to detect and respond to environmental cues essential for its cellular development, survival, and virulence [25]. HKs serve as integral components in sensing and transmitting signals associated with antibiotic exposure, enabling the bacterium to initiate downstream responses aimed at evading or neutralizing the antibiotic threat [26]. Specifically, this enzyme belongs to the transferase type and is situated in the cytoplasmic membrane (Figure S1A). It responds to stimuli originating from the periplasm by undergoing autophosphorylation of a histidine residue, which subsequently transmits a signal to an ATP-binding kinase domain.

Table 2 provides the functional characterization of these proteins using the KEGG database and the subcellular localization data from a consensus of two servers (PSORT and CELLO). Remarkably, excluding HK, the remaining 11 proteins belong to an efflux pump family comprising 3 different proteins: an outer porin-like protein called Outer Membrane Factor (OMF, in light green in Figure S1B) that spans the outer membrane in gram-negative bacteria, an inner membrane protein receiving the family name Resistance/Nodulation/cell Division (RND, in gray in Figure S1B), and a periplasmic protein named Membrane Fusion Protein (MFP, in dark green in Figure S1B) that connects the two previous proteins. These antiporter multidrug efflux pumps belong to the RND family [27,28]. The RND efflux pump systems are universally found across almost all biological kingdoms, utilizing common mechanisms that greatly enhance the diversity of resistance profiles [29].

The proposed functional mechanisms entail a coordinated rotation of the pump components, transitioning through three consecutive states: site access, substrate binding, and extrusion [29]. It has been reported that dynamic phenomena involved in the substrate binding and the subsequent extrusion differ from those observed in an inhibitor-bound state, where rotational movement is blocked, thus impeding the extrusion [30]. The main proteins forming the MFP group include MexA, MexX, MexC, and MexE; for the RND type, we found: MexB, MexY, MexD, and MexF; and OMF proteins comprise OprM, OprJ, and OprN proteins [31]. The MexAB-OprM, MexXY-OprM, MexCD-OprJ, and MexEF-OprN pumps, which belong to the RND superfamily, are linked to resistance against most antipseudomonal medications when overexpressed [32]. The MexCD-OprJ operon in *P. aeruginosa* is usually expressed at low levels, but it plays a crucial role in expelling various antibiotics and toxic substances, including quinolones, chloramphenicol, and tetracyclines [33]. The MexEF-OprN system typically remains inactive under standard conditions. However, its overexpression has been associated with resistance to chloramphenicol, quinolones, trimethoprim, and certain β-lactams [34]. Both the MexCD-OprJ and MexEF-OprN efflux systems play a role in quorum sensing (QS) by expelling signal molecules such as 4-hydroxy-2-heptylquinoline and kynurenine. This reduces the accumulation of QS signals within the cell, thereby influencing the regulation of QS-controlled virulence factors [35]. The MexXY efflux pump is one of the primary resistance mechanisms in *P. aeruginosa* but lacks a coding sequence for an OMF. However, it functions as a multidrug efflux pump in conjunction with OprM from the MexAB-OprM operon [36]. Like other RND pumps, MexXY is essential for resistance to aminoglycosides, fluoroquinolones, and certain β-lactams [37]. The MexAB-OprM operon, recognized as the first multidrug efflux pump in *P. aeruginosa*, is the major contributor to antibiotic resistance [38]. Increased expression of the MexAB-OprM efflux pump is linked to resistance against almost 50 antibiotics [29].

The identity percentages for OMF proteins (Figure S2A) and MFP proteins (Figure S2B) were determined for both canonical proteins and the 11 proteins studied. Among these, 7 proteins (Q9HXB9, Q9HU26, Q9I0V8, Q9HY88, Q9I006, Q9HWH3, and Q9I0Y7) were classified as OMF proteins, while the other 4 proteins (Q9I0V5, Q9I0Y9, Q9I3R2, and G3XD25) were categorized as the MFP type. In terms of OMF proteins, only two exhibit more than 35% identity with canonical proteins: Q9HWH3, with 37.10% identity to OprN, and Q9I0Y7, with 96.61% identity to OprN protein. Meanwhile, among the MFP proteins, Q9I0Y9 demonstrates 99.03% identity to MexE, and G3XD25 displays 71.00% identity to MexC. These identity percentages provide insights into the structural similarities between the studied proteins and their canonical counterparts, aiding in the elucidation of their functional roles and evolutionary relationships.

### 2.5. Predicting and Refining the Three-Dimensional Structure and Multimeric Assembly in RND Proteins

**Figure 3:**
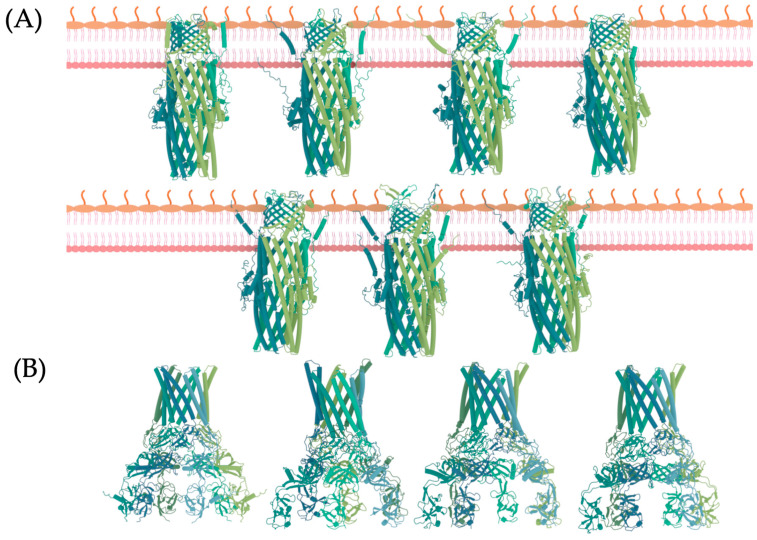
Three-dimensional structures of the 11 proteins were modeled in the study. (**A**) Outer Membrane Factors (OMF)-type proteins. (**B**) Membrane Fusion Protein (MFP)-type proteins. The membrane model represents the outer membrane of Gram-negative bacteria. Each color represents a distinct monomer of the protein, with three monomers in OMF and four in MFP proteins.

In the subsequent filtering step, the HK protein is discarded to narrow down the focus on conducting drug repositioning for the 11 proteins of the RND efflux pump. Upon conducting pairwise alignment of our sequences against the RCSB-Protein Data Bank (PDB) database [39], it was observed that only the Q9I0Y7 protein of the OMF type has been resolved through experimental methods. Specifically, it was resolved using the X-ray diffraction method, yielding a resolution of 1.69 Å. The corresponding PDB code for this resolved structure is 5ZAP [40] (Table S2). We used the AlphaFold server [41] to predict their 3D structures for the remaining proteins. AlphaFold is an AI system developed by DeepMind, capable of accurately predicting the 3D structures of proteins. It achieves this through the application of deep learning algorithms trained on extensive datasets comprising protein sequences and their corresponding 3D structures. However, its current version typically predicts monomeric forms of proteins and not the multimeric complexes that RND proteins form in their functional states. Table S2 in our study presents the sequences of the proteins under investigation, along with the corresponding PDB code and the percentage of identity of their closest homologs with resolved 3D structures and assembled multimeric units. This information served as input for constructing the multimers of each protein through structural alignments and replication of each subunit. Specifically, OMF type proteins are composed of three identical subunits, forming homotrimers. On the other hand, MFP proteins typically form homohexamers, as depicted in Figure 3. To mitigate steric impediments arising from multimerization, we conducted energy minimization and 50 ns NPT MD simulations for each protein. This process involved optimizing the atomic positions of the multimeric structures to relieve any potential clashes or overlaps and achieve more stable conformations.

### 2.6. Rescuing Relevant Protein Conformations and Repositioning Drugs through Ensemble-docking and Non-Covalent Interactions

Ensemble-docking enhances the accuracy of virtual screening by considering multiple conformational states of the target protein, reflecting its dynamic nature and improving the identification of effective drug candidates [42]. In this study, we employed ensemble-docking combined with various docking energy-based metrics. Utilizing multiple metrics allows for a more robust selection process, as it mitigates the biases and limitations associated with relying on a single metric [43,44]. The three components of the OprM-MexAB pump, derived from the 6TA6 crystallographic structure, were employed as protein controls. Furthermore, a set of 8 known RND pump inhibitors served as ligand controls: P9D (also called ABI-PP or D13-9001) [45,46], cefiderocol (CEF) [29], doxorubicin (DM2) [46,47,48], minocycline (MIY) [45,46,48], oxacillin [29,46,49], MBX [45], rhodamine-6G [29,46,47], and erythromycin [29,45,46]. The components were simulated separately for 50 ns, and a root mean square deviation (RMSD, Figure S3) dendrogram was calculated for each, resulting in 5 clusters per component. Subsequently, a representative frame from each cluster was selected, and 5712 FDA-approved drugs were repositioned, along with the ligand controls. Given that most RND pump inhibitors have been reported to interact primarily with the internal component of the pump (MexB). We docked the ligand controls into the MexB binding site. Then, we computed the median binding energy for all conformations of MexB for each ligand control to identify the most affine inhibitor. This inhibitor was used as a benchmark in subsequent docking evaluations of the other proteins under study. Among the 8 inhibitors tested, P9D, CEF, and DM2 showed the strongest binding affinities to MexB, as depicted in Figure S4. Notably, P9D in its two tautomeric forms (P9D_1 and P9D_2) had the lowest median energy.

Remarkably, the binding energy trend for P9D, CEF, and DM2 persisted with the OprM and MexA controls (Figure S4), albeit with binding energies approximately 2 kcal/mol higher than those observed with MexB. Additionally, among the three components of the RND pump, the internal protein (MexB) consistently demonstrated superior binding energies with all the repositioned compounds (FDA dataset). Based on these findings, we selected P9D as the control ligand for the remaining evaluations, using its median binding energy in each protein as the cutoff criterion.

**Figure 4:**
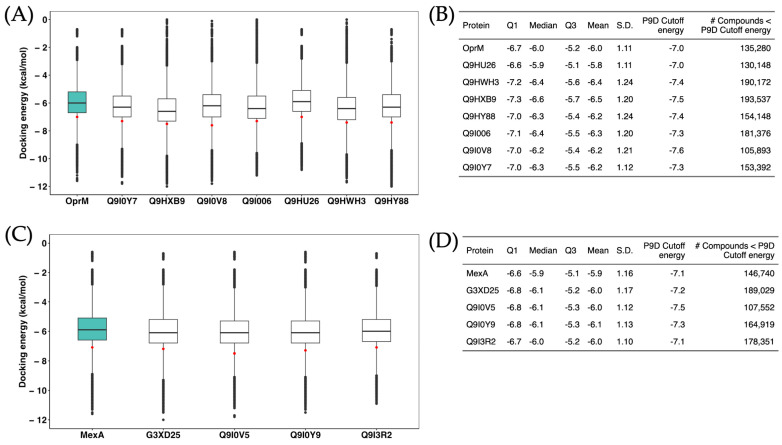
Population of docking energies. (**A**) The boxplots depict the distribution of docking poses for the 5712 drugs repositioned against each of the OMF-type proteins. Within each boxplot, the red dot represents the median docking energy for the control inhibitor (P9D), serving as a reference. Additionally, the green boxplot represents the control protein (OprM) used in the study for comparative analysis. (**B**) This section presents the descriptive statistical analysis of the docking energy values obtained for each protein. It includes the first quartile (Q1), median, third quartile (Q3), average, and standard deviation (S.D.) of the docking energies. Furthermore, it highlights the median docking energy value for the control inhibitor (P9D), akin to the red dot in section (**A**). Additionally, it provides the number of compounds exhibiting docking energy values better than the control used as cutoff. (**C**,**D**) These sections mirror sections **A** and **B** but focus on MFP-type proteins, respectively, using the MexA protein as control (the green boxplot). Notate that OprM and MexA data are the same showed in Figure S4.

In our ensemble-docking protocol for drug repositioning with the 11 proteins under study, we observed consistent trends for both OMF and MFP type proteins. For OMF proteins, the repositioned compounds exhibited affinity energies comparable to those of the control protein (OprM), with a median energy around −6.0 kcal/mol (Figure 4A). Moreover, the data dispersion was consistent across all proteins. The median energy of the most effective known inhibitor, used as a cutoff, was approximately −7.0 kcal/mol for both OprM and the other seven OMF group proteins. Notably, for each protein, we identified over 100,000 repositioned drugs with binding affinity energies surpassing the median affinity energy of the control inhibitor (Figure 4B). Similarly, for MFP type proteins (Figure 4C), we observed a trend parallel to that of the control protein (MexA), with median energies around −6.0 kcal/mol and comparable data dispersions across all cases. Additionally, we identified over 100,000 repositioned drugs with binding affinity energies more favorable than the median energy of the control inhibitor (around −7.0 kcal/mol, Figure 4D).

The total energy population of our study is calculated by multiplying the number of proteins (p), frames (f), docking grids (g), docking poses (dp), and repositioned drugs (c). This calculation yields 5,997,600 docking values for the OMF proteins (7 × 5 × 3 × 10 × 5712) and 4,569,600 docking values for the MFP type proteins (4 × 5 × 4 × 10 × 5712), resulting in more than 10 million molecular docking evaluations in total. When selecting the best inhibitor candidates, we applied multiple criteria to the energetic populations of docked molecules [50]. For each repositioned drug, we considered all docking poses obtained from each protein conformation and each docking run (with varying grid positions). We calculated several metrics: the top-scoring energy (the most negative value), the median energy, and the arithmetic, geometric, and harmonic means. Each drug was then ranked based on these individual metrics. To determine the overall ranking, we calculated the mode of these rankings. For instance, if a compound consistently ranks first across several of the five metrics, its mode would be 1, indicating it as a top candidate. Additionally, we assessed the frequency of these rankings; if a compound ranks first in three out of the five metrics, its frequency is 3/5. The goal was to select candidates that ranked highest in at least 3 out of the 5 metrics and were present in at least 60% of the proteins evaluated (6 out of 11). This rigorous approach allowed us to robustly identify the most promising inhibitors.

We identified three compounds as the top candidates across the majority of the evaluated proteins:

- MK-3207: This molecule is currently undergoing phase 2 clinical trials and is associated with migraine disorders [51]. It acts as an antagonist of the Calcitonin gene-related peptide type 1 receptor in humans. MK-3207 ranks first in the rankings for all MFP type proteins and in 6 out of 7 OMF proteins.
- R-428 (Bemcentinib): This compound, in clinical phase 2, is being investigated for various pathologies, including myelodysplastic syndrome, melanoma, acute myeloid leukemia, and mesothelioma. It acts by inhibiting the Tyrosine-protein kinase receptor [52]. R-428 is the top-ranked compound across all metrics for 100% of the proteins evaluated.
- Suramin: Currently advancing to phase 3 of clinical development, Suramin is associated with the treatment of non-small cell lung carcinoma, prostate adenocarcinoma, autism spectrum disorder, and acute kidney injury. This compound functions as an acidic fibroblast growth factor inhibitor [53,54]. Suramin appears as an inhibitor in all MFP proteins and 4 out of 7 OMF type proteins.

**Figure 5:**
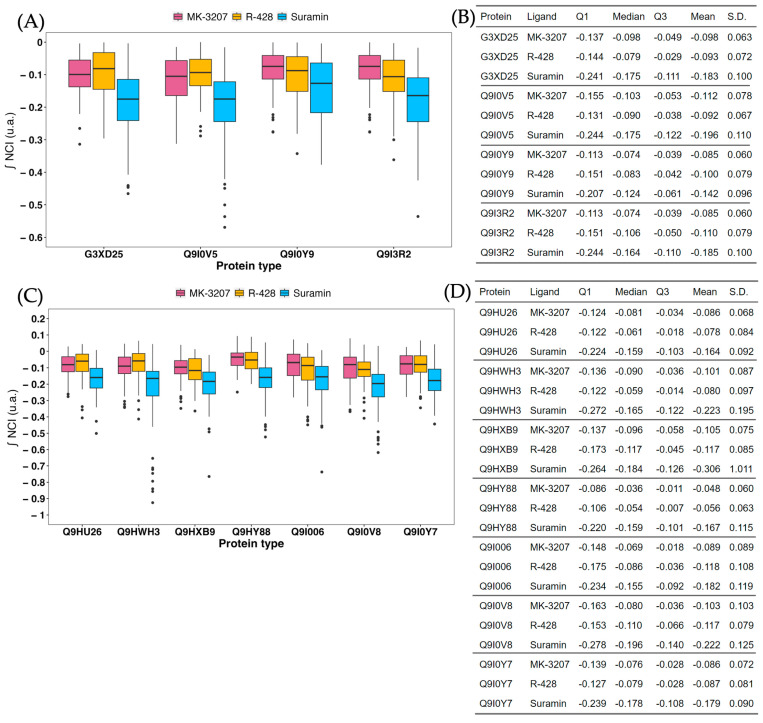
Promolecular energy distribution calculations for the top 3 drug candidates. (**A**) Energy distribution was calculated from the integral of the Non-Covalent Interactions (NCI) indices for all docking poses obtained for the top 3 candidates: MK-3207 (pink), R-428 (yellow), and Suramin (blue) against all OMF-type proteins. The NCI integral provides an energy value, with more negative values indicating stronger protein–ligand interactions. (**B**) Descriptive statistics for each OMF-type protein–ligand pair, including the first quartile (Q1), median, third quartile (Q3), average, and standard deviation (S.D.) of the affinity energy. (**C**) Energy distribution similar to (**A**), for the top 3 candidates against all MFP-type proteins. (**D**) Descriptive statistics for each MFP-type protein–ligand pair, including Q1, median, Q3, average, and S.D. of the affinity energy.

The final stage of our filtering process involved evaluating the three best inhibitor candidates for each protein studied using the non-covalent interaction index method (Figure 5 and Table S3). This method provides a more accurate quantification of all electronic effects involved in the protein–ligand interaction compared to traditional docking methods. Overall, we observed that the compound Suramin consistently exhibits better attractive energies across all evaluated proteins compared to MK-3207 and R-428.

## 3. Materials and Methods

### 3.1. Subtractive Proteomics

The proteomic datasets for the *P. aeruginosa* PAO1 reference strain (ATCC: 15692; UniProt ID: UP000002438) with 5564 predicted proteins and *Homo sapiens* (UniProt ID: UP000005640) with 79,684 proteins were retrieved from the UniProt repository [55] (www.uniprot.org). To enhance the quality of the datasets, we implemented an additional step in our pipeline aimed at eliminating redundant information, specifically duplicated proteins. To achieve this, we employed CD-Hit software v.4.8.1 [56] (https://sites.google.com/view/cd-hit, accessed on 30 December 2023) and OrthoMCL software v.2.0 [57] (https://orthomcl.org/orthomcl/app, accessed on 30 December 2023). These tools were applied to the *P. aeruginosa* proteome, ensuring the removal of paralogous sequences by retaining only one copy of each protein. The criteria for eliminating homologous proteins included an amino acid identity greater than 60% and an e-value exceeding 10^−5^. Additionally, protein sequences shorter than 100 amino acids were excluded from the final dataset. This refined approach enhanced the accuracy and reliability of the proteomic dataset generated in this work, providing a concise protein subset for subsequent analyses.

### 3.2. Search for Essential Protein Sequences of P. aeruginosa with Low Similarity to the Human Proteome and Human Bacterial Microbiome

Since the objective of our study is to repurpose drugs for proteins identified through the subtractive proteomics process, to ensure the safety and efficacy of these repurposed drugs, it is crucial to avoid potential side effects that may arise from the drugs binding to homologous human proteins. Therefore, we excluded proteins with high homology to the human proteome and the proteomes in the human microbiota. The screening process used BLAST+ protein software v.2.15.1 [58] (https://blast.ncbi.nlm.nih.gov/, accessed on 10 January 2024) installed locally. This software was employed to identify homologous sequences between the proteomes from *P. aeruginosa* and humans, subsequently excluding them from the dataset. To ensure the specificity of the analysis, a cutoff criterion was set, requiring a bit score value greater than 100 and an e-value exceeding 10^−5^.

The human gut, known for harboring a diverse community of approximately 1000 microorganisms, plays a crucial role in maintaining health. In consideration of the microbial landscape, a strategic filtering process was implemented to prevent potential cross-interactions between molecular targets of *P. aeruginosa* and the vast array of microorganisms constituting the human bacterial microbiome. To execute this, we utilized the BLAST+ protein software. The objective was to filter out homologous sequences from the *P. aeruginosa* proteome by comparing them against the extensive repertoire of the human bacterial microbiome. Our reference dataset consisted of 299,611 proteins curated from 73 bacteria commonly found in the gut (Table S1), as reported by Shanmugham and Pan [59]. It eliminated homologous sequences between the *P. aeruginosa* proteome and the human microbiome available, employing stringent cutoff criteria—a bit score value exceeding 100 and an e-value greater than 10^−5^. In the final step of the first filter of our protocol, we searched for essential proteins in *P. aeruginosa* through analysis using the DEG Database [60] (Database of Essential Genes, http://origin.tubic.org/deg/public/index.php/search/bacteria, accessed on 10 January 2024). It used *P. aeruginosa* PAO1 as the reference genome (RefSeq: NC_002516), which encompasses 117 essential genes; the analysis involved a comparison with our protein dataset obtained from the preceding steps. A cutoff was set at a bit score value greater than 100 and an e-value exceeding 10^−10^.

### 3.3. Characterizing Pharmacological Targets in P. aeruginosa: Integrating Virulence, Resistance, and Antigenicity Analyses

To identify suitable pharmacological targets among the 116 essential proteins of *P. aeruginosa*, we conducted a comprehensive analysis focusing on three critical characteristics: virulence, resistance, and antigenicity. This approach ensures that the selected targets are not only essential for bacterial survival but also relevant for therapeutic intervention.

(a) **Virulence Factors:** We used the Virulence Factor Database (VFDB) [61] (http://www.mgc.ac.cn/VFs/, accessed on 10 January 2024) to identify proteins involved in virulence. Virulence factors are crucial for the pathogenicity of *P. aeruginosa*, making them ideal targets for drug development. We applied stringent criteria with a cutoff set at a bit score value greater than 100 and an e-value exceeding 10^−5^;
(b) **Resistance Factors:** Resistance factors were identified using the BacMet2 database, including both experimental and predicted data [62] (http://bacmet.biomedicine.gu.se/, accessed on 10 January 2024). Resistance mechanisms enable *P. aeruginosa* to withstand antibiotic treatments, so targeting these factors can enhance the efficacy of therapeutic strategies. We employed the same stringent cutoff criteria (bit score > 100, e-value < 10^−5^) to accurately detect resistance-related proteins;
(c) **Antigenicity Features:** The antigenicity of essential proteins was predicted using VaxiJen [63] (https://www.ddg-pharmfac.net/vaxijen/VaxiJen/VaxiJen.html, accessed on 10 January 2024). This tool assesses the potential of proteins to elicit an immune response, which is vital for the development of vaccines and immunotherapies. Identifying antigenic proteins ensures that our targets can be used to stimulate protective immunity against *P. aeruginosa* infections.

### 3.4. Identifying Pharmacological Candidates for P. aeruginosa Essential Proteins

The 23 sequences that meet the criteria of virulence, bacterial resistance, and antigenicity were further assessed for their druggability using the DrugBank Database [64] (https://go.drugbank.com/, accessed on 30 January 2024). This database served as a comprehensive resource for assessing whether each essential protein identified in our pipeline is recognized as a viable druggable target in *P. aeruginosa* or other microorganisms by the FDA.

The primary focus of our search was on drugs capable of inhibiting the activity of specific essential proteins in *P. aeruginosa.* To validate the uniqueness of each target, the protein sequences were submitted to the DrugBank web server for comparison with existing druggable targets. The filters applied included considering only approved targets and specifying protein types as targets, enzymes, carriers, or transporters.

### 3.5. Characterizing the Biological Classification of P. aeruginosa Essential Proteins

The remaining 11 sequences capable of meeting all the previously evaluated criteria were analyzed for biological characterization:

(a) **Subcellular localization:** The PSORT v.3.0 [65] (https://www.psort.org/psortb/, accessed on 2 March 2024) and CELLO2GO [66] (http://cello.life.nctu.edu.tw/cello2go/, https://www.psort.org/psortb/, accessed on 2 March 2024) databases were employed to determine the subcellular localization of the proteins.
(b) **Functional classification:** The KEGG [67] (Kyoto Encyclopedia of Genes and Genomes, https://www.genome.jp/kegg/pathway.html, https://www.psort.org/psortb/, accessed on 2 March 2024) database was used to classify the proteins into functional biological categories.

### 3.6. Predicting 3D Structures and Supramolecular Assembly of Essential Proteins in P. aeruginosa

The filtration process identified 11 essential proteins necessitating the prediction of their three-dimensional (3D) structures to facilitate inhibitor identification. Utilizing their sequences, a BLAST search against the PDB database was conducted. For proteins lacking experimental structural data (NMR, X-ray, or Cryo-EM) in the PDB, the AlphaFold server was employed to predict their structures based on amino acid sequences. Once the structures were determined, an extensive review of the literature was conducted to elucidate their assembly patterns, whether as monomers, dimers, trimers, or higher-order supramolecular complexes. Additionally, BLAST searches against the PDB database were performed to identify structurally resolved proteins closely related to our proteins of interest. These homologous structures served as templates for three-dimensional alignment, aiding in the construction of supramolecular assemblies by replicating and aligning each subunit based on the templates to form functional multimeric complexes.

### 3.7. Refining 3D Structures through Molecular Dynamics Simulations

To enhance the accuracy and stability of the predicted 3D structures, MD simulations were conducted. This computational approach enables the exploration of protein dynamics over time, facilitating the attainment of energetically favorable conformations. In our simulations, proteins were immersed in a periodic boundary box of pre-equilibrated Single Point Charge (SPC) water molecules and neutralized with sodium chloride, ensuring an appropriate physiological environment. For membrane proteins, they were embedded within a patch of pre-equilibrated 1-palmitoyl-2-oleoyl-sn-glycero-3-phosphocholine (POPC) membrane at 300 K before the addition of water molecules. Each system underwent thermodynamic equilibration following a protocol similar to that described by Bustos et al. [68], implemented using the Maestro/Schrodinger suite [69]. Subsequently, a 50 ns production simulation was conducted under an NPT ensemble (T = 300 K and P = 1 atm), using the OPLS3e force field [70]. Notably, no energetic constraints were applied to the protein during the simulation, allowing it to energetically relax solely based on environmental constraints.

### 3.8. Identification of Relevant Protein Conformations and Drug Repurposing through Ensemble-docking and Non-Covalent Interaction Index

To explore diverse protein conformations and facilitate drug repurposing, each MD trajectory was analyzed using the Bio3D library [71] in R to generate dendrograms based on RMSD. The cutree() function was employed to partition the trajectories into 5 clusters. From each cluster, 5 representative frames were randomly selected and converted into the PDBQT format with the Open Babel tool [72].

Subsequently, a collection of over 7000 FDA-approved, clinical trials, and pre-clinical compounds from various pathologies was obtained from the CLUE.io database [73]. These compounds underwent preprocessing using the Ligprep tool in the Maestro/Schrodinger suite to ensure proper ionization, stereochemistry, and 3D structure optimization. The resulting set comprised 5712 compounds after excluding cyclic peptides and accounting for different tautomeric and protonation states at pH 7.0. These compounds were further converted to PDBQT format using the Open Babel tool [72]. For each MFP protein, four different grids (32 Å × 32 Å × 32 Å) were generated in each frame, positioned at distinct internal regions. Similarly, for OMF proteins, three grids per frame were generated. These grids served as docking sites for the 5712 compounds, with 10 poses collected *per* compound, grid, and protein. This exhaustive docking procedure generated more than 2.5 million docking outputs for subsequent analysis.

To serve as a control, we utilized the tridimensional structure of MexAB-OprM from *P. aeruginosa*, obtained via electron microscopy at a resolution of 3.20 Å (PDB id: 6TA6 [74]). Additionally, we selected eight known RND pump inhibitors: P9D, CEF, DM2, MIY, oxacillin, MBX, rhodamine-6G, and erythromycin as ligand controls. The tripartite pump was separated into its three components (MexA, MexB, and OprM), and we conducted the same MD simulations, frame selections, and ensemble-docking protocol previously described for the studied proteins.

We computed the ranking of the eight ligand controls in MexB based on their median binding affinities. Once the top three ligand controls were identified, these were used as cutoffs in the docking evaluations for the 11 proteins under study, as well as for the MexA and OprM components. The top three median docking energies of these selected inhibitors served as benchmarks to evaluate the binding affinities of the 5712 drugs in the proteins studied.

The dockings were run with the VINA GPU implementation [75]. AutoDock Vina employs an empirical scoring function to estimate the binding affinity between ligands and proteins. This scoring function is inspired by the X-Score function [76] and includes terms for steric interactions, hydrophobic interactions, and hydrogen bonding [77]. Specifically, it combines attractive Gaussian and repulsive parabolic functions for steric interactions, linear functions for hydrophobic interactions, and piecewise linear functions for hydrogen bonding. Each term is weighted and summed to predict the overall binding affinity. The search algorithm used by Vina is an Iterated Local Search global optimization algorithm, which involves generating random initial conformations, performing mutations followed by local optimization.

Selection of the most promising inhibitor candidates was conducted using custom R scripts, which integrated various metrics such as minima (top scoring), arithmetic, geometric, and harmonic means, and medians. This approach, inspired by the methodology described by Yáñez et al. [50], aimed to identify energetically favorable interactions between compounds and proteins across the large population of data, ensuring robust inhibitor selection tailored to each protein under investigation.

To complement the extensive exploration carried out on molecular docking, approximately 4000 integration calculations were performed on the NCI regions for protein–ligand interactions [78]. These calculations provided detailed insight into the non-covalent forces involved in protein–ligand binding, including hydrogen bonds, hydrophobic interactions, and other types of intermolecular interactions. To perform the NCI calculation, the promolecular approximation was employed, which provides a description of the approximate electron density constructed by linearly combining the densities of the individual atoms/fragments that make up the molecular system, without considering their mutual polarization effects. The use of promolecular density allows NCI analysis to be applied to very large systems, such as proteins or nucleic acids, where a full quantum calculation is too computationally expensive. The promolecular approach provides a reasonable approximation of the true electron density at a much lower computational cost. This approach allowed an efficient and reliable evaluation of the characteristics of the non-covalent interactions in the protein–ligand complex, thus facilitating the interpretation of the molecular docking results and providing valuable information for the rational design of new bioactive compounds.

This analysis consists of deriving analytical expressions for the NCI equations and integrating them in the NCI regions to obtain closed-form solutions for the binding energies. Details of the approach used for this work are given below:

The NCI equations relate properties of the electron density (*ρ*) and its derivatives like the reduced density gradient (*s*) and the density Hessian eigenvalues (*λ_i_*) to identify and characterize non-covalent interaction regions. Analytical expressions can be derived for quantities like the volume (V) and integrated density (*q*) of the attractive (*λ*_2_ < 0) and repulsive (*λ*_2_ > 0) NCI regions in terms of *ρ*, *s*, *λ_i_*, etc. [79]

(1) $$q_{att}^{n}=\int_{\Omega (NCI)}\rho^{n}\left(\overset{⃑}{r}\right)\;d\overset{⃑}{r}\;\lambda_{2}\left(\overset{⃑}{r}\right)<0$$

(2) $$q_{rep}^{n}=\int_{\Omega (NCI)}\rho^{n}\left(\overset{⃑}{r}\right)\;d\overset{⃑}{r}\;\lambda_{2}\left(\overset{⃑}{r}\right)>0$$

(3) $$q_{bind}^{n}=-\left(q_{att}^{n}-q_{rep}^{n}\right)$$

- These analytical expressions for *q* can then be integrated over the NCI regions to obtain closed-form solutions relating them to the intermolecular separation;
- The binding energy can be approximated as a functional of $q_{att}^{n}-q_{rep}^{n}$, and fit to reproduce known binding curves, e.g., $E_{bind}=f(q_{att}^{n}-q_{rep}^{n})$ where n is an optimized exponent;
- The key advantage is obtaining analytical, transferable expressions for $E_{bind}$ in terms of NCI region properties that are valid over the entire potential energy surface.

This fully analytical approach aims to directly relate binding energies to real-space properties of the electron density within the NCI regions through derived closed-form expressions. All these calculations have been carried out with the NCIplot V4.2 software (https://github.com/juliacontrerasgarcia/NCIPLOT-4.2, accessed on 10 January 2024) [80,81].

The mechanisms of action of the control inhibitors used in our study are well-documented. For instance, MBX2319 targets the AcrB efflux pump in *E. coli* by binding to the “hydrophobic trap” within the deep substrate binding pocket, inhibiting the pump’s function by preventing necessary conformational changes and hindering substrate binding [45]. D13-9001 (P9D) specifically inhibits the MexAB-OprM efflux pump in *P. aeruginosa* by binding to a unique site near the deep substrate binding pocket, preventing conformational changes required for pump function [46]. Both AcrB and MexB use a peristaltic mechanism powered by the proton motive force to transport substrates, and inhibitors like MBX2319 and P9D disrupt their function by binding to specific sites, enhancing antibiotic activity [48]. Our study, however, focuses on the upper two-thirds of the RND efflux pumps. Given this focus, it is challenging to directly identify the mechanisms of action, as the well-characterized mechanisms of the control inhibitors occur predominantly in the lower third of the pumps. Despite this, the relevance of our study lies in its emphasis on the less studied upper regions of the efflux pumps, opening new research avenues. We speculate that our identified molecules may obstruct the conduction pore or inhibit the assembly of the pump. Although this research is in its early stages, our identified inhibitor candidates exhibit high affinity across all proteins studied, presenting two significant advantages: (1) These compounds demonstrate high multi-target or broad-spectrum activity within the RND family, despite the studied proteins sharing a low percentage of identity. (2) They bind with high affinity not only to one component of the pump but to all three components, suggesting multiple potential modulatory sites within the channel.

## 4. Conclusions

In this study, we employed a comprehensive approach to identify and characterize essential proteins in *P. aeruginosa* (PAO1 strain), elucidating their potential as pharmacological targets.

Our analysis revealed 12 essential proteins, including a histidine kinase and 11 members of the RND efflux pump family (OMF and MFP proteins), crucial for coordinating resistance mechanisms and pathogenicity. By predicting their three-dimensional structures and multimeric assemblies, we facilitated the identification of potential inhibitor candidates through molecular docking simulations. Notably, we identified MK-3207, R-428, and Suramin as promising inhibitor candidates across multiple metrics, demonstrating their potential efficacy against a range of pathologies.

Furthermore, our study leveraged non-covalent interaction index methods to refine the selection of inhibitor candidates, providing insights into the precise electronic effects involved in protein–ligand interactions. Suramin consistently exhibited superior attractive energies across all evaluated proteins compared to MK-3207 and R-428, suggesting its potential as a broad-spectrum inhibitor.

The newly identified drug candidates, selected through a repurposing strategy, are already FDA-approved for other indications. This provides an advantage as these drugs have established pharmacokinetic and pharmacodynamic profiles, ensuring their efficacy in human patients. Candidates such as MK-3207, R-428 (Bemcentinib), and Suramin have shown promising binding affinities against the RND efflux pumps of *P. aeruginosa*, suggesting their potential effectiveness in combating multidrug-resistant strains by targeting and modulating efflux pump functions. Additionally, the safety profiles of these drugs are well-documented, reducing the risk of unforeseen adverse effects. Our study further ensures specificity by excluding targets with high homology to human proteins, minimizing potential off-target interactions and cross-reactivity. Overall, our findings contribute to the understanding of essential proteins in *P. aeruginosa* and provide a framework for the rational design of novel therapeutic interventions targeting bacterial virulence and resistance mechanisms.

The accuracy of the subtractive proteomics approach heavily depends on the comprehensiveness and accuracy of the databases used (e.g., VFDB, BacMet2, VaxiJen, and DEG). Any gaps or inaccuracies in these databases could affect the identification of virulence, resistance, and antigenic factors. Ensemble docking relies on a limited number of conformational states (five in our study). This may not fully capture the dynamic nature of the proteins, potentially overlooking some critical conformations that could affect binding affinity predictions. Given these limitations, experimental validation of our findings would be highly beneficial. Conducting in vitro and in vivo assays would confirm the computational predictions and provide more reliable insights into the efficacy of the identified drug candidates. Such validation steps are crucial to bridge the gap between computational predictions and practical therapeutic applications, ensuring that the proposed drug candidates are both effective and safe for use.

## Figures and Tables

**Table 1:** Subtractive proteomics analysis using *P. aeruginosa* proteins for finding relevant pharmacological targets. This table outlines the sequential filtering steps applied to the *P. aeruginosa* proteome to isolate essential proteins suitable for drug targeting. Each step details the number of proteins selected and discarded, refining the dataset to identify 116 essential proteins as potential pharmacological targets.

Protocol	Number of Proteins Selected	Number of Proteins Discarded
Total of proteins (*P. aeruginsa* proteome)	5564	0
Remove paralogs sequences	5453	111
Remove sequences lower than 100 amino acids	5120	333
Remove sequences homologous to human proteom	4344	776
Remove sequences orthologous to microbiota in humans	3360	984
Identification of proteins essential for bacterial survival	116	3244

**Table 2:** Functional characterization of *P. aeruginosa* essential proteins identified in our pipeline, categorized into Histidine Kinase, MFP-type proteins, and OMF-type proteins from the RND family.

Uniprot ID	KEGG Orthology	KEGG Additional Information		Subcellular Localization Consensus
Q9HTZ0	Two-component system (Histidine Kinase)	Enzyme (Transferases)	Signal transduction	Cytoplasmatic Membrane
Q9I0Y7	Outer Membrane Factor (Multidrug Efflux System)	Transporters	Antimicrobial resistance genes	Outer Membrane
Q9HXB9 *				
Q9I0V8 *				
Q9I006 *				
Q9HU26 *				
Q9HWH3				
Q9HY88				
Q9I0V5	Membrane Fussion Protein (Multidrug Efflux System)	Transporters	Antimicrobial resistance genes	Cytoplasmatic Membrane
Q9I0Y9				
Q9I3R2				
G3XD25				

* Hypothetical protein into PAO1 genome. The subcellular localization is a consensus of PSORT and CELLO servers.

## Data Availability

The original contributions presented in the study are included in the article and Supplementary Materials.

## Supplementary Materials

The supporting information can be downloaded at: https://www.mdpi.com/article/10.3390/ijms25158027/s1.
