# Supplementary material for: From Proteome to Potential Drugs: Integration of Subtractive Proteomics and Ensemble Docking for Drug Repurposing against Pseudomonas aeruginosa RND Superfamily Proteins
Source: Int J Mol Sci. 2024 Jul 23;25(15):8027. doi: 10.3390/ijms25158027 (PMC11312079; doi:10.3390/ijms25158027)
Supplement: Supplementary file 1 [file ijms-25-08027-s001.zip › ijms-3052883-supplementary.pdf]

International Journal of Molecular Sciences

**Table S1.** List of bacteria commonly found in the human gut microbiome. This list is derived from Shanmugham and Pan [59], detailing various microorganisms and their respective strains.

| # | Microorganism | Strain |
|---|---|---|
| 1 | Actinomy ces odontolyticus | ATCC 17982 |
| 2 | Akkermansia muciniphila | ATCC BAA-835 |
| 3 | Alistipes putredinis | DSM 17216 |
| 4 | Anaerofustis stercorihominis | DSM 17244 |
| 5 | Anaerostipes caccae | DSM 14662 |
| 6 | Anaerotruncus colihominis | DSM 17241 |
| 7 | Pseudoflavonifractor capillosus | ATCC 29799 |
| 8 | Bacteroides cellulosilyticus | DSM 14838 |
| 9 | Bacteroides coprocola | DSM 17136 |
| 10 | Bacteroides dorei | DSM 17855 |
| 11 | Bacteroides eggerthii | DSM 20697 |
| 12 | Bacteroides finegoldii | DSM 17565 |
| 13 | Bacteroides intestinalis | DSM 17393 |
| 14 | Bacteroides ovatus | ATCC 8483 |
| 15 | Bacteroides pectinophilus | ATCC 43243 |
| 16 | Bacteroides plebeius | DSM 17135 |
| 17 | Bacteroides stercoris | ATCC 43183 |
| 18 | Bacteroides uniformis | ATCC 8492 |
| 19 | Bifidobacterium adolescentis | ATCC 15703 |
| 20 | Bifidobacterium adolescentis | L2-32 |
| 21 | Bifidobacterium angulatum | DSM 20098 |
| 22 | Bifidobacterium bifidum | DSM 20456 |
| 23 | Bifidobacterium breve | DSM 20213 |
| 24 | Bifidobacterium dentium | ATCC 27678 |
| 25 | Bifidobacterium longum | DJO10A |
| 26 | Bifidobacterium longum | NCC2705 |
| 27 | Bifidobacterium longum subsp infantis | str ATCC 15697 |
| 28 | Borrelia burgdorferi | CA-11.2A |
| 29 | Butyrivibrio crossotus | DSM 2876 |
| 30 | Catenibacterium mitsuokai | DSM 15897 |
| 31 | Clostridium asparagiforme | DSM 15981 |
| 32 | Clostridium bartlettii | DSM 16795 |
| 33 | Clostridium bolteae | ATCC BAA-613 |
| 34 | Clostridium hiranonis | DSM 13275 |
| 35 | Clostridium leptum | DSM 753 |
| 36 | Clostridium methylpentosum | DSM 5476 |
| 37 | Clostridium nexile | DSM 1787 |
| 38 | Clostridium ramosum | DSM 1402 |
| 39 | Clostridium scindens | ATCC 35704 |
| 40 | Clostridium | sp L2-50 |
| 41 | Clostridium | sp M62/1 |
| 42 | Clostridium | sp SS2/1 |
| 43 | Clostridium spiroforme | DSM 1552 |
| 44 | Clostridium sporogenes | ATCC 15579 |
| 45 | Clostridium symbiosum | ATCC 14940 |
| 46 | Collinsella aerofaciens | ATCC 25986 |
| 47 | Collinsella intestinalis | DSM 13280 |
| 48 | Collinsella stercoris | DSM 13279 |
| 49 | Coprococcus comes | ATCC 27758 |
| 50 | Coprococcus eutactus | ATCC 27759 |
| 51 | Dorea formicigenerans | ATCC 27755 |
| 52 | Dorea longicatena | DSM 13814 |
| 53 | Eggerthella lenta | DSM 2243 |
| 54 | Enterobacter cancerogenus | ATCC 35316 |
| 55 | Eubacterium dolichum | DSM 3991 |
| 56 | Eubacterium hallii | DSM 3353 |

| | | |
|---|---|---|
| 57 | Eubacterium siraeum | DSM 15702 |
| 58 | Eubacterium ventriosum | ATCC 27560 |
| 59 | Faecalibacterium prausnitzii | A2-165 |
| 60 | Faecalibacterium prausnitzii | M21/2 |
| 61 | Lactobacillus salivarius | UCC118 |
| 62 | Methanobrevibacter smithii | ATCC 35061 |
| 63 | Methanobrevibacter smithii | DSM 11975 |
| 64 | Methanobrevibacter smithii | DSM 2374 |
| 65 | Methanobrevibacter smithii | DSM 2375 |
| 66 | Mitsuokella multacida | DSM 20544 |
| 67 | Parabacteroides johnsonii | - |
| 68 | Parabacteroides merdae | ATCC 43184 |
| 69 | Parvimonas micra | ATCC 33270 |
| 60 | Photorhabdus luminescens | subsp laumondii TTO1 |
| 61 | Prevotella copri | DSM 18205 |
| 62 | Providencia alcalifaciens | DSM 30120 |
| 63 | Providencia rettgeri | DSM 1131 |
| 64 | Providencia rustigianii | DSM 4541 |
| 65 | Roseburia faecis | M72/1 |
| 66 | Roseburia intestinalis | L1-82 |
| 67 | Ruminococcus gnavus | ATCC 29149 |
| 68 | Ruminococcus lactaris | ATCC 29176 |
| 69 | Ruminococcus obeum | ATCC 29174 |
| 70 | Ruminococcus torques | ATCC 27756 |
| 71 | Streptococcus infantarius subsp infantarius | ATCC BAA-102 |
| 72 | Subdoligranulum variabile | DSM 15176 |
| 73 | Victivallis vadensis | ATCC BAA-548 |

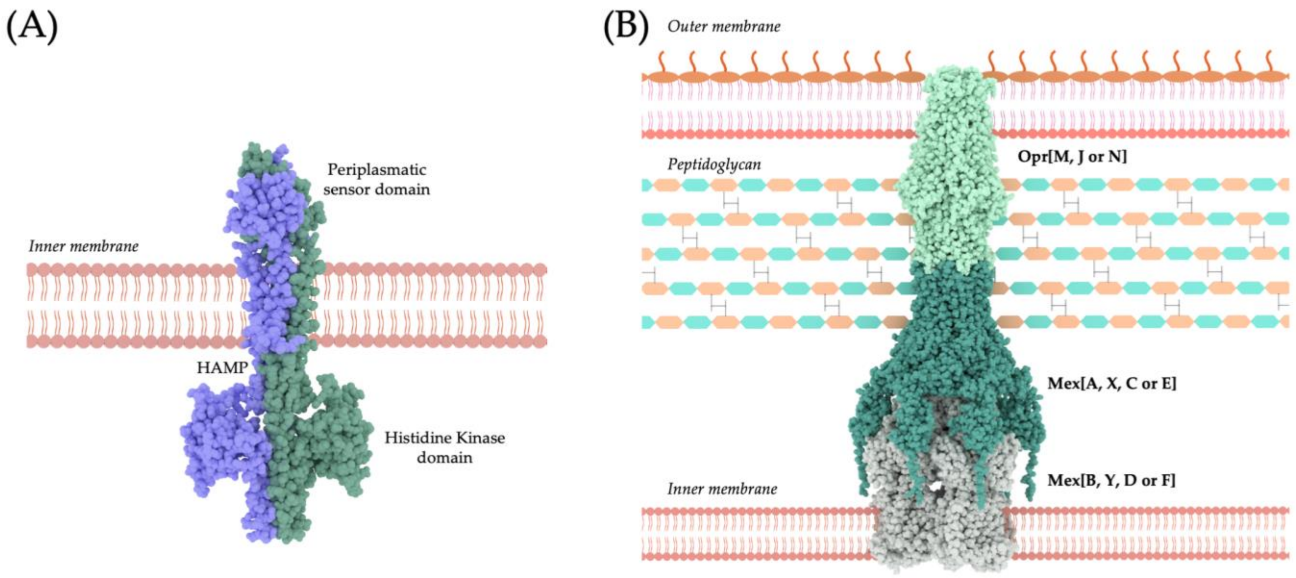


**Figure S1.** Types of essential proteins selected in the subtractive proteomics stage. This figure presents the three-dimensional structures of essential proteins that met all criteria used in the subtractive proteomics pipeline. A) Histidine Kinase, embedded in the inner membrane. B) A tripartite RND efflux pump, shown with its components spanning the inner membrane, peptidoglycan layer, and outer membrane.

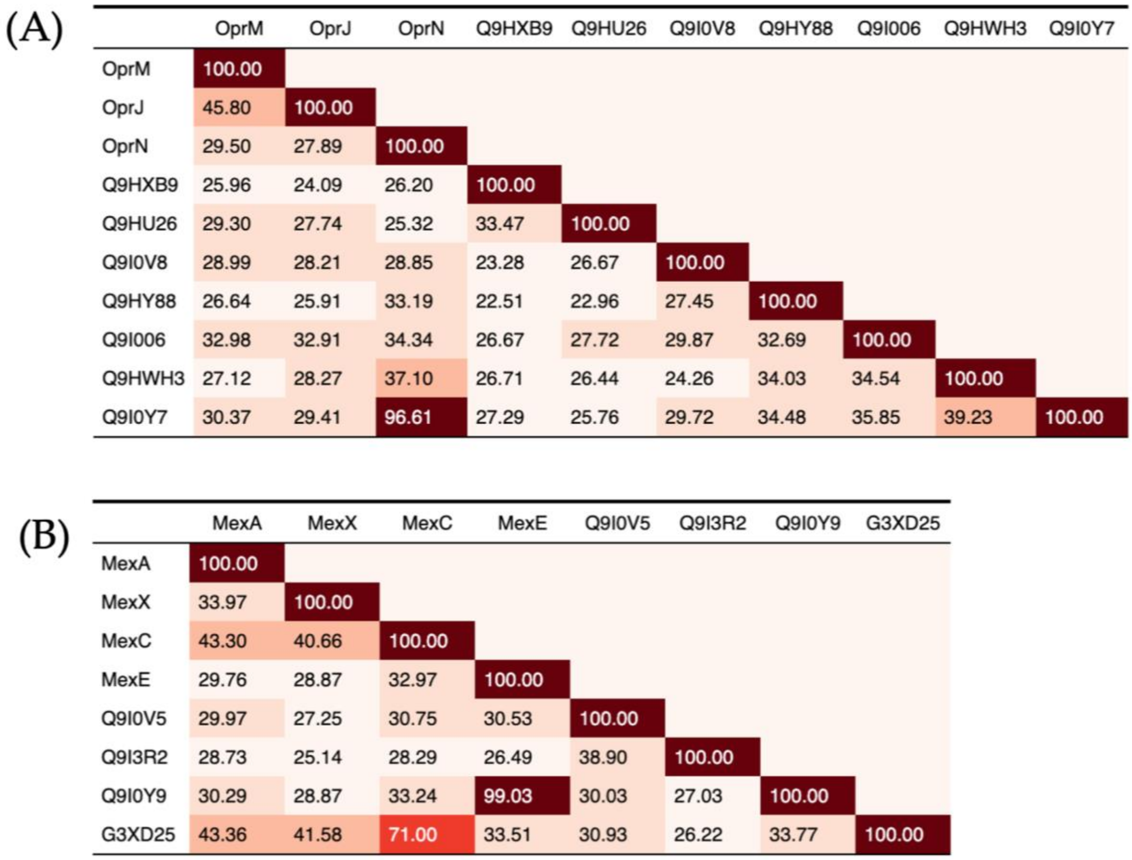


**Figure S2.** Sequence identity heatmap of the studied proteins. A) OMF-type proteins studied and the canonical OprM, OprJ and OprN proteins. B) MFP-type proteins studied and the canonical MexA, MexX, MexC and MexE proteins. The darker the color, the higher the sequence identity of the compared protein pair.

**Table S2. Selected RND proteins from the complete protocol for drug repurposing.** RND proteins, detailing their UniProt ID, the closest matching structure available in the Protein Data Bank (PDB), the percentage of sequence identity against the PDB protein, and their amino acid sequences for both OMF and MFP proteins.

| Protein Type | Uniprot protein accession ID | PDB ID of most related protein | % sequence identity of the closest protein | Aminoacid sequence |
|---|---|---|---|---|
| Outer Membrane Factor | Q9I0Y7 | 5AZP | 100% | MIHAQSIRSGLASALGLFSLLALSACTVGPDYRTPDTAAAKIDAT ASKPYDRSRFESLWWKQFDDPTLNQLVEQSLSGNRDLRVAFARL RAARALRDDVANDRFPVVTSRASADIGKGQQPGVTEDRVNSER YDLGLDSAWELDLFGRIRRQLESSDALSEAAEADLQQLQVSLIAE LVDAYGQLRGAQLREKIALSNLENQKESRQLTEQLRDAGVGAEL DVLRADARLAATAASVPQLQAEAERARHRIATLLGQRPEELTVD LSPRDLPAITKALPIGDPGELLRRRPDIRAAERRLAASTADVGVAT ADLFPRVSLSGFLGFTAGRGSQIGSSAARAWSVGPSISWAAFDLG SVRARLRGAKADADAALASYEQQVLLALEESANAFSDYGKRQE RLVSLVRQSEASRAAAQQAAIRYREGTTDFLVLLDAEREQLSAED AQAQAEVELYRGIVAIYRSLGGGWQPSA |

| | | | | |
|---|---|---|---|---|
| | Q9HXB9 | 1YC9 | 36,73% | MVGSFVGFLVVFSAISGCVSTGDIAPEAATLDANALATDHAIQA<br>AAREAGWPQAQWWKVYADPQLDAWIEKALDGNPGLAVAHA<br>RVRQAKSMAGLVESIESPQIEGKGSLVRHRWPDDYFYGPGDLAR<br>TTSWNNSTEIGLNYKLDLWGRDRSDSERAVDLAHMAAAEARQ<br>AQLELEGNIVRAYVQLSLQYAEMDIAKAMLQQQRDILALAQRR<br>LRGGIGTHFEVSQAEVPLPETERRIEVIDEEIQLTRNLLAALAGKG<br>PGEGRTIRRPSLNLAAQPSLPSALPAELLGRRPDVVARRWQVAA<br>LAKGVDVARADFYPNVDLMASVGFSAVGGGMLEFFRSAKYTYS<br>AGPAVTLPIFDGGRLRSQLGEAAAGYDAAVEQYNQTLVDALKN<br>ISDQLIRLHSVDIQKDFAAQSVASAQKTYDIATLAYQRGLTDYLN<br>VLNAQTRLFQQQLVQEQVQAARLAAHASLLTALGGGVGAGAD<br>TPAQRKLAPENVPVRAVSSR |
| | Q9I0V8 | 1WP1 | 28.87% | MKHTPSLLALALVAALGGCAIGPDYQRPDLAVPAEFKEAEGWR<br>RAEPRDVFQRGAWWELYGDQTLNDLQMHLERSNQTLAQSVAQ<br>FRQAEALVRGARAAFFPSITGNVGKTRSGQGGGDSTVLLPGGST<br>VSSGGSGAISTSYSTNLSVSWEVDLWGKLRRQLEANQASLHASA<br>ADLAAVRLSQQSQLAQNYLQLRVMDEQIRLLNDTVTAYERSLK<br>VAENKYRAGIVTRADVAQARTQLKSTQAQAIDLKYQRAQLEHA<br>IAVLVGLPPAQFNLPPVASVPKLPDLPAVVPSQLLERRPDIASAER<br>KVISANAQIGVAKAAYFPDLTLSAAGGYRSGSLSNWISTPNRFWS<br>IGPQFAMTLFDGGLIGSQVDQAEATYDQTVATYRQTVLDGFREV<br>EDYLVQLSVLDEESGVQREALESAREALRLAENQYKAGTVDYTD<br>VVTNQATALSNERTVLTLLGSRLTASVQLIAAMGGGWDSADIER<br>TDERLGRVEEGLPPSP |
| | Q9I006 | 5IUY | 36.44% | MKGTPLLLIASLALGACSLGPDFTRPDRPAPGEWSLQAAAGNPS<br>HLAAAPLAAQWWTLFDDAQLNALLQRVQRANLDLRSAAARL<br>QQSRAIRRSLGGDALPSVDASGNYQRQRTTSAGLFDPSGKAGKG<br>NYNHALAGFDASWELDFWGRVRRELEAADATVEASENELRDV<br>QVSVLAEAARDYIQLRGEQNRAAIIRDNLETARRSLELTRTRLAN<br>GVATDLEVAQALAQVASMEARLPEVEKNQAHLVNALGLVGAS<br>PGSLLAELGPARAIPRPPGSVPVGLPSELAQRRPDIRRAEARLHA<br>ATASIGVAKADFYPRITLNGNFGFESLQLSSLGDWDHRQFAIGPA<br>FSLPIFEGGRLRGRLELREAQQQEAAIDYQRTVLRAQEVDDAMH<br>DYAANQRRQERLGEAVAQNRRALQSAREQYRAGAVDFLSVLDS<br>QRQLLDNQEQQVASDEAVSLTLVNLYKALGGGWSPTSDPASG |
| | Q9HU26 | 1YC9 | 36.90% | MPFPLLHPWPQRLALASAILLAAGCVTSEGLEPNARLQPAGALQ<br>AGRSLDGVALSPAAWPRQDWWTGLGDRQLDQLIGEALQGTPD<br>LQIAEARARQAAATAQAQDAARQPTLDAKASYSGIRAPTSVAP<br>APLGGRYSAIKYLSLGFNYDFDLWGGERAAWEAALGQANAARI<br>DSQAARIGLSASIARAYSDLAHAFTVRDLAEEELKRSQRMTELSQ<br>KRMSAGLDSKVQLQQTQTQLATARQQLSAAEQDIASARIALAV<br>LLGKGPDRGLELQRPQPLNPASLSLPSVLPAELLGRRADIVAAR<br>WRVEAARRNIDSAKTEFYPNLNLGAMAGLAALHTSDVLQAPSR<br>FFQVAPAISLPIFDGGRRRANLAERDADYDLAVGQYNKTLVQAL<br>GEVSDDLGKLRSLEQQVIDQRQARDIARSNFDLAMRRYGEGVGS<br>YLDALSVQQQLLVAERQLASLESQQIDLSVQLVQALGGGFQPDS<br>RSAALATAKAPAE |
| | Q9HWH3 | 5IUY | 39.86% | MKRSYPNLSRLALALAVGTGLAACSVGPDYQRPQSPPPRVASEH<br>LGEFSGERREAPWWSFFDDPQLVRLVDQALARNHDIREARANL<br>RSARALFDDRWLDQLPQVTSQAGYSRSIEQQLDYDGEPRRRLAE<br>SYRAGFDAQWEIDLFGRLGRLSDAALARAEAADADLRLVRLSIA<br>ADTARAYFEIQGYQRRLDVARAQVRSWRDTLELTRSSLQLGSGL<br>PEDVENAQANLLRSEAAIPPLTTALESARYRLDVLRGEAPGSGAP<br>ILDGGAAAPLAKNLPLGDVDRLILQRPDVVSAERQLAASTEDVG<br>AATAELYPRLDLGGFIGFFALRSGDLGSASRAFELAPSVSWPAFR<br>LGNVRARLRAVEAQSDAALARYQRSLLLAQEDVGNALNQLAE<br>HQRRLVALFQSATHGANALEIANERYRAGAGSYLAVLENQRAL<br>YQIREELAQAETASFVNVIALYKALGWGSGDLAPGAGQLAAGE<br>TAGANR |
| | Q9HY88 | 5AZO | 35.57% | MKPYLRSSLSALILLGGCAAVGPDYAPPSASAPASFGAMPAGIDG<br>SGVEIEWWRGFDEPALESLIQRALAANLDIALAGARLDEAKALL<br>RENREEFLPRGGPAFDYQARRRGEVETPAGQQRDIETYRGALDA<br>SWEIDLFGRVRRSVEAAEAQAGSREALLRNVQASVAATVAMSW<br>FQLQGIEAELAVVHDIAGNQRDSLEMVERLVSAGSAHEFDRLRA<br>EALLHNVEAAVPDLERRRAATRNALAVLLAEAPQAFSPPVARA<br>SGERLTLRTLGVGDPAGLLARRADIAAAERNLAAATARIGVETA<br>GLYPQVEVRGSIGLVAGNLDALDESGTSFNVLNPVIRWALLDRG<br>RVWARIAASEARAQEALILYDRTVLRALQETDDAFNGYGAAAD<br>RLRLRLLEATANREAARLARERFVQGDGEYLDVLEAERSDYLSR<br>RALSIARTEQRLAVVGIYKALGGGWEACAGARRCGVATDDTSP<br>GVARQRDSRS |
| Membrane Fussion Protein | Q9I0V5 | 5N5G | 33.33% | MTPTTGKSKFRTLRPWLITALAFAAVIGLVMWLAAPASAPSSDG<br>RPGRGGKPGAALPKANALTVGVARVEQGDLALHFNALGTVTA<br>FNTVNVKPRVNGELVKVLFQEGQEVKAGDLLAVVDPRTYKAAL<br>AQAEGTLMQNQAQLKNAEIDLQRYKGLYAEDSIAKQTLDTQEA<br>QVRQLQGTIRTNQGQVDDARLNLTFTEVRAPISGRLGLRQVDIG<br>NLVTSGDTTPLVVITQVKPISVVFSLPQQQIGTVVEQMNGPGKLT<br>VTALDRNQDKVLAEGTLTTLDNQIDTTTGTVKLKARFENADGK |

| | | | | |
|---|---|---|---|---|
| | | | | LFPNQFVNVRLLAQTLKGVLTIPANAVQRGTNGIYVYVVGADN<br>KVSQRSVAIGTSENERVVVESGLKAGEQVVVEGTDRLRDGMEVR<br>VAEASPQVLEGEPQKPQTGRPSGLQGDSVGSGSAE |
| | Q9I0Y9 | 5V5S | 34.53% | MEQSSHFSWRYPLALAAVLVLSACGKAPETTQGMAAPKVSVAE<br>VIEQPLNEWDEFTGRLEAPESVELRPRVSGYIDRVAFHEGALVKK<br>GDLLFQIDPRPFEAEVKRLEAQLQQARAAQARSVNEAQRGERLR<br>ASNAISAELADARTTAAQEAKAAVAATQAQLDAARLNLSFTRIT<br>APIDGRVSRAEVTAGNLVNSGETLLTTLVSTDKVYAYFDADERV<br>FLKYVELARQAGRDTRSESPVYLGLSSEDGNPHLGRLDFLDNQV<br>NPRTGTIRGRAVFDNAKGEFTPGLYVRLKLVGSKTYAATLIKDEA<br>VGTDLGKKFVLVLDGDNKTVYRTVEMGPKLEGLRIVRSGLSKGD<br>RIVVNGLQRVRPGMQVDPQKVEMASADTLATLARLRQSVGDSE<br>PPKVAASKDNATRNEPRG |
| | Q9I3R2 | 5V5S | 32.09% | MQALRSGGGRVLVGVLAAGLVAFGGWAWLGGDAGAKAAPAP<br>ARVPVIVARVERRDVEQQV<br>SGIGTVTSLHNVVIRTQIDGQLTRLLVSEGQMVEAGELLATIDDR<br>AVVAALEQAQASRASNQAQLKSAEQDLQRYRSLYAERAVSRQL<br>LDQQQATVDQLRATLKANDATINAERVRLSYTRITSPVSGKVGIR<br>NVDVGNLVRVGDSLGLFSVTQIAPISVVFSLQQEQLLQLQALLGG<br>EAAVRAYSRDGGSALGEGRLLTIDNQIDSSTGTIRVRASFDNRQA<br>RLWPGQFVAVSLHTGVRRDQLVLSSKAVRRGLEGNFVYRVADD<br>RVEAVPVRVLQDIDGLSVVEGLASGDQVVVDGHSRLMPGALVD<br>IQEPRPSLAQATERRP |
| | G3XD25 | 5V5S | 46.82% | MADLRAIGRIGALAMAIALAGCGPAEERQEAAEMVLPVEVLTV<br>QAEPLALSSELPGRIEPVRVAEVRARVAGIVVRKRFEEGADVKAG<br>DLLFQIDPAPLKAAVSRAEGELARNRAVLFEAQARVRRYEPLVKI<br>QAVSQQDFDTATADLRSAEAATRSAQADLETARLNLGYASVTA<br>PISGRIGRALVTEGALVGQGEATLMARIQQLDPIYADFTQTAAEA<br>LRLRDALKKGTLAAGDSQALTLRVEGTPYERQGALQFADVAVD<br>RGTGQIALRGKFANPDGVLLPGMYVRVRTPQGIDNQAILVPQR<br>AVHRSSDGSAQVMVVGADERAESRSVGTGVMQGSRWQITEGLE<br>PGDRVIVGGLAAVQPGVKIVPKPDGAQAQAQSPAPQQ |

(A)

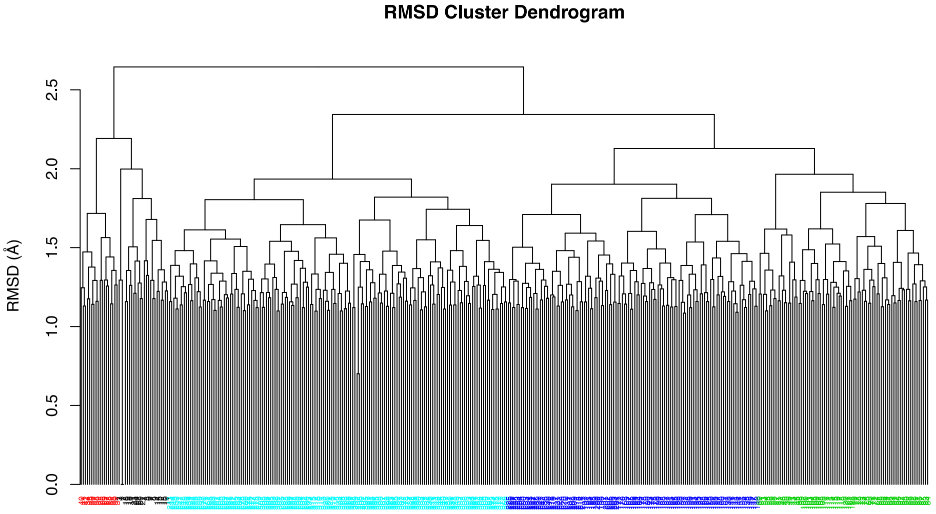
RMSD Cluster Dendrogram
RMSD (Å)
0.0
0.5
1.0
1.5
2.0
2.5

(B)

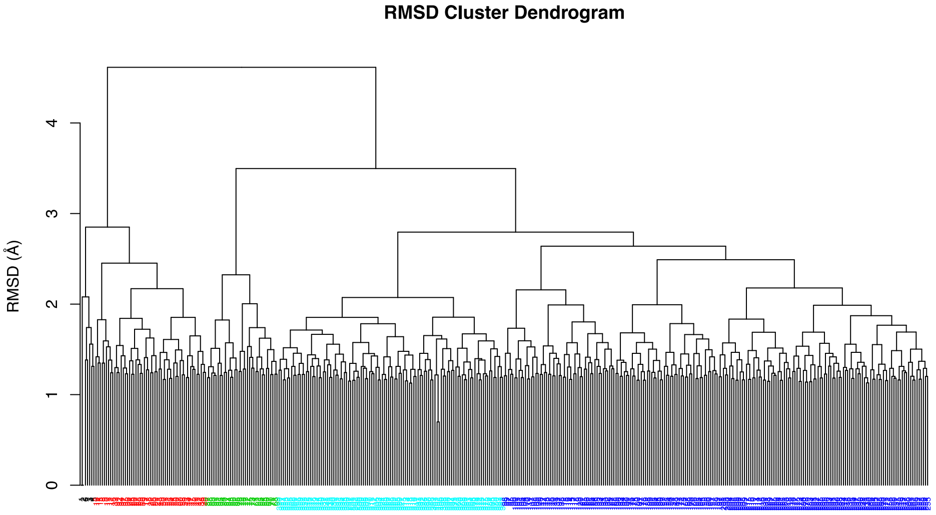
RMSD Cluster Dendrogram
RMSD (Å)
0
1
2
3
4

(C)

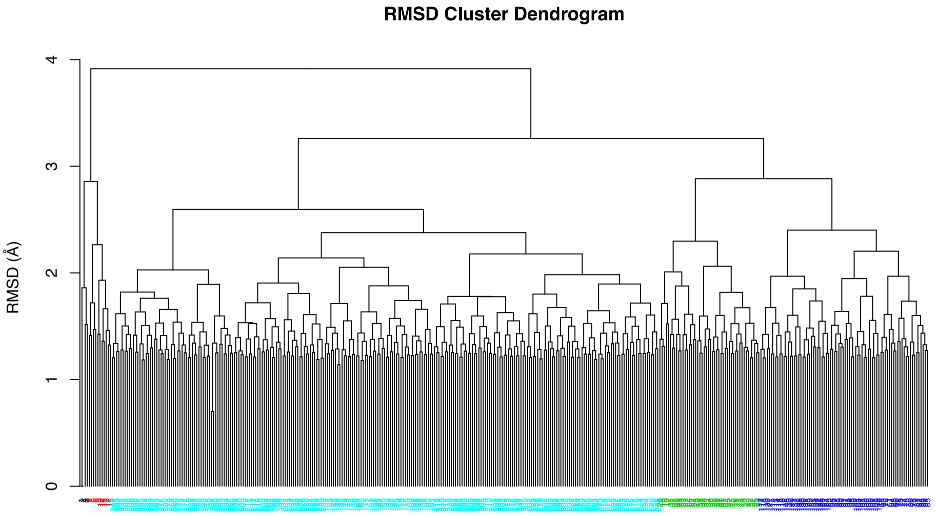
RMSD Cluster Dendrogram
RMSD (Å)
0
1
2
3
4

(D)

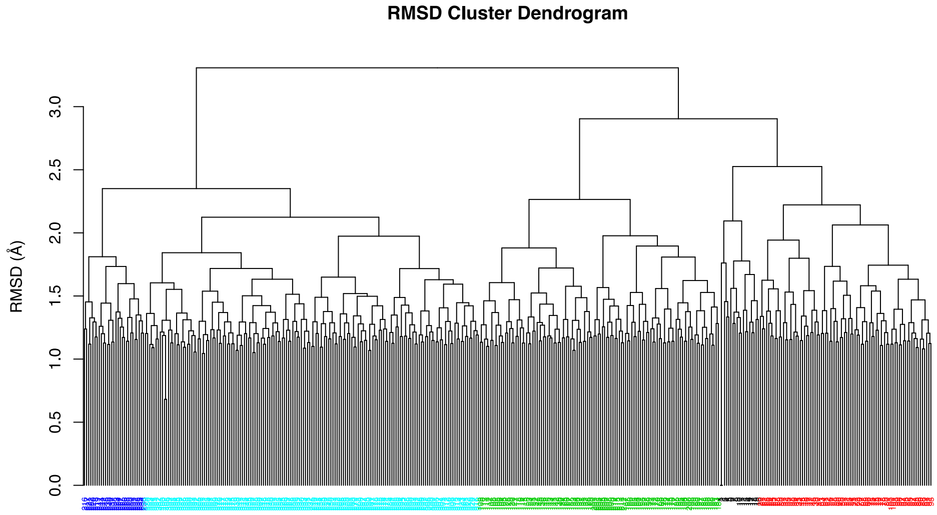
RMSD Cluster Dendrogram
RMSD (Å)
0.0
0.5
1.0
1.5
2.0
2.5
3.0

(E)

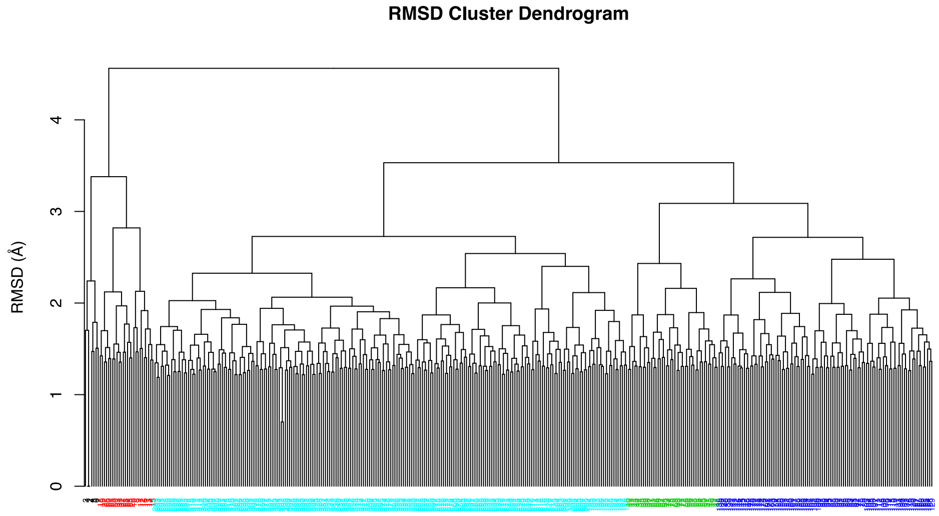
RMSD Cluster Dendrogram
RMSD (Å)
0
1
2
3
4

(F)

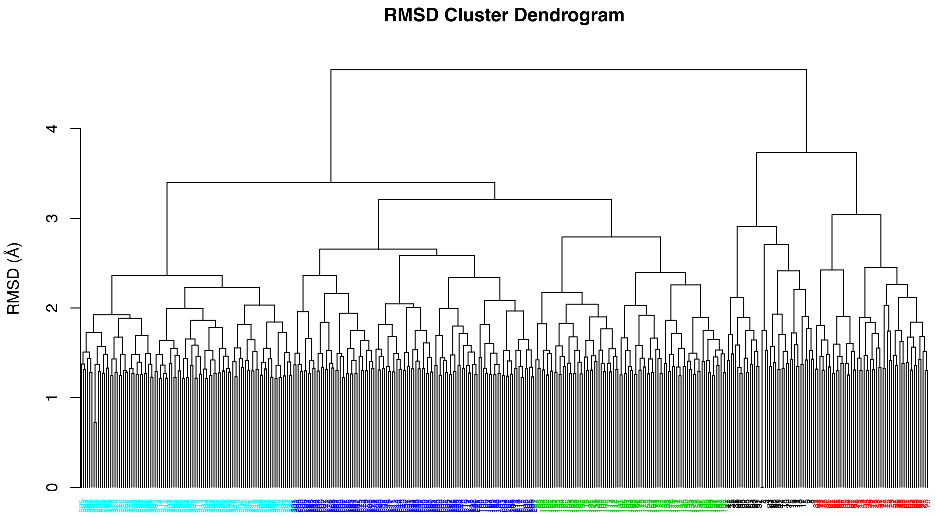
RMSD Cluster Dendrogram
RMSD (Å)
0
1
2
3
4

(G)

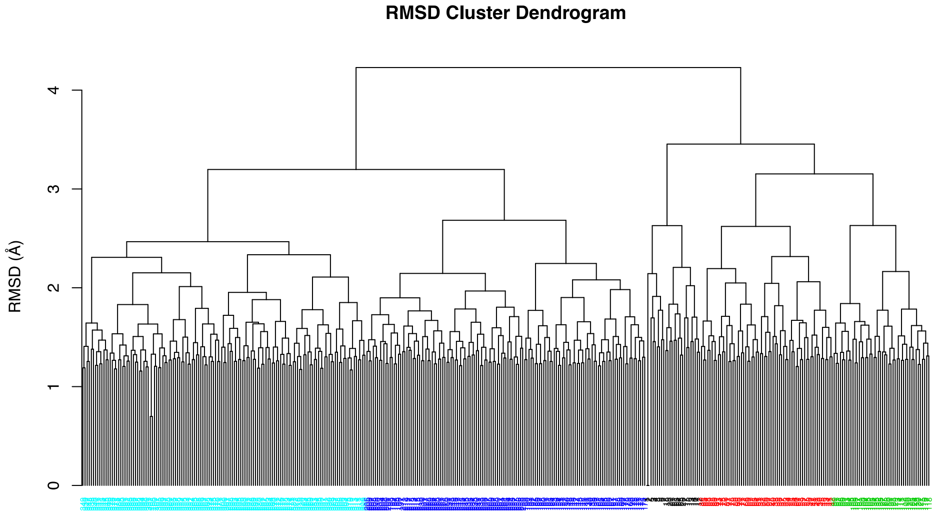
RMSD Cluster Dendrogram
RMSD (Å)
0
1
2
3
4

(H)

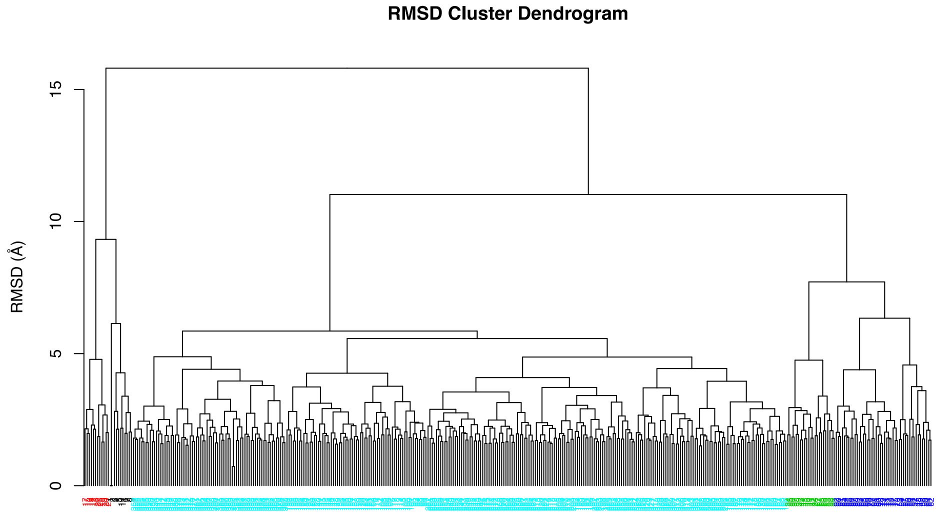
RMSD Cluster Dendrogram
RMSD (Å)
0
5
10
15

(I)

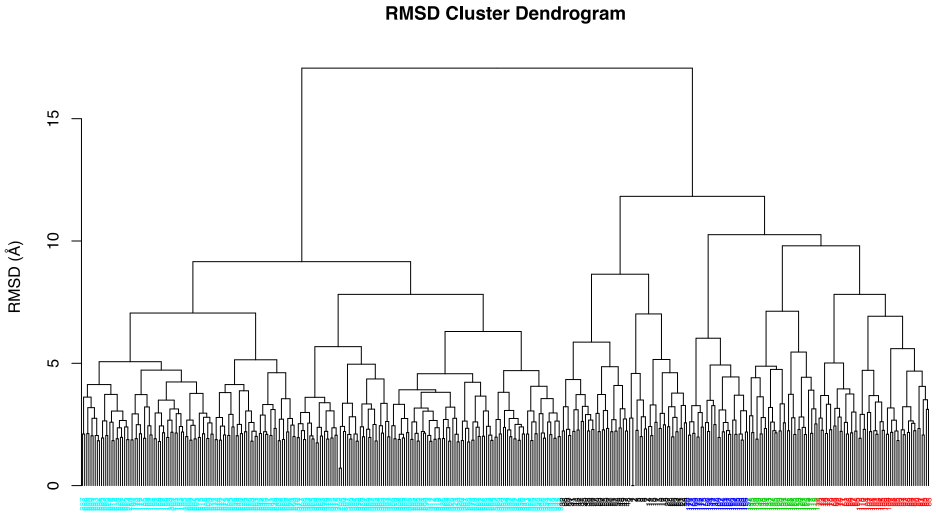
RMSD Cluster Dendrogram
RMSD (Å)
0
5
10
15

(J)

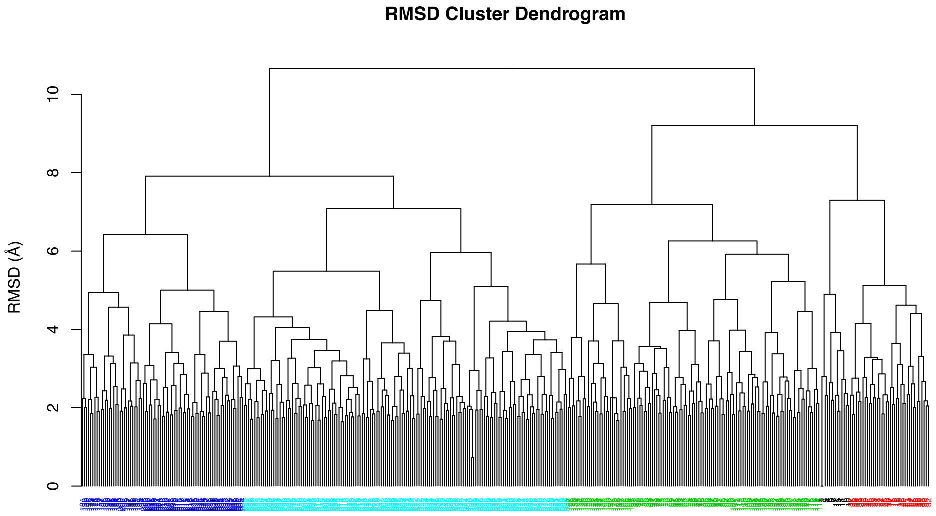
RMSD Cluster Dendrogram
RMSD (Å)
0
2
4
6
8
10

(K)

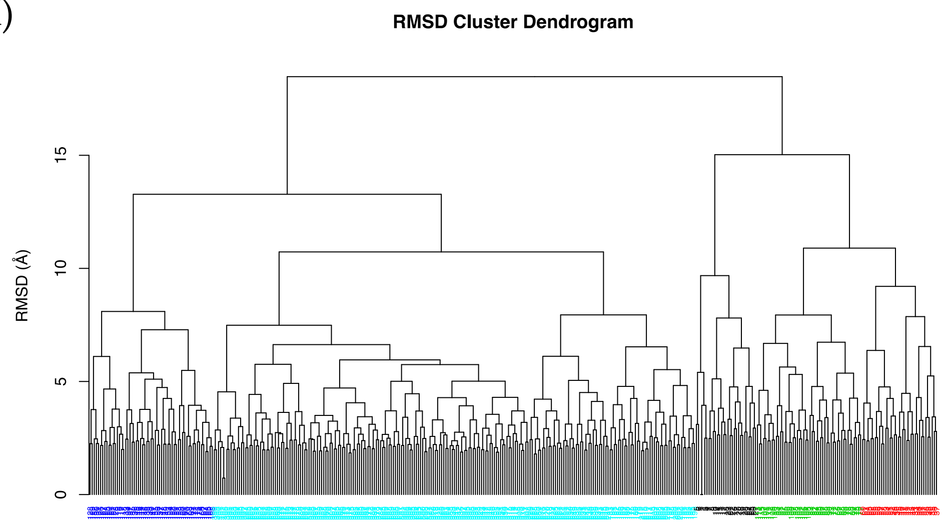
RMSD Cluster Dendrogram
RMSD (Å)
0
5
10
15

(L)

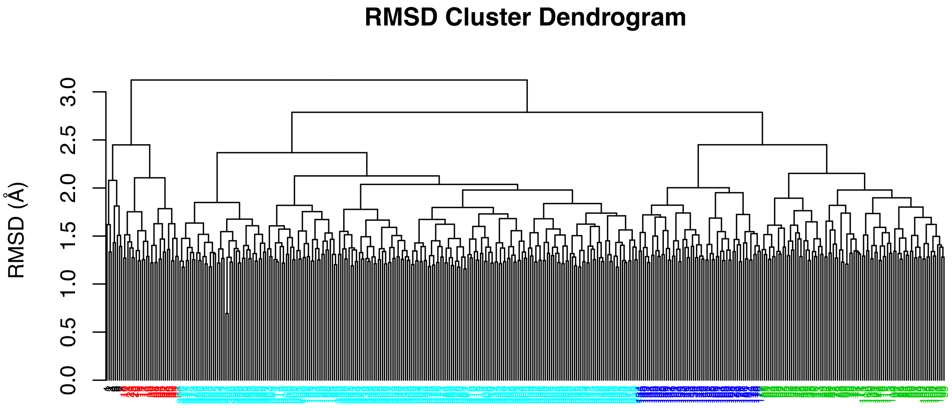
RMSD Cluster Dendrogram
RMSD (Å)
0.0
0.5
1.0
1.5
2.0
2.5
3.0

(M)

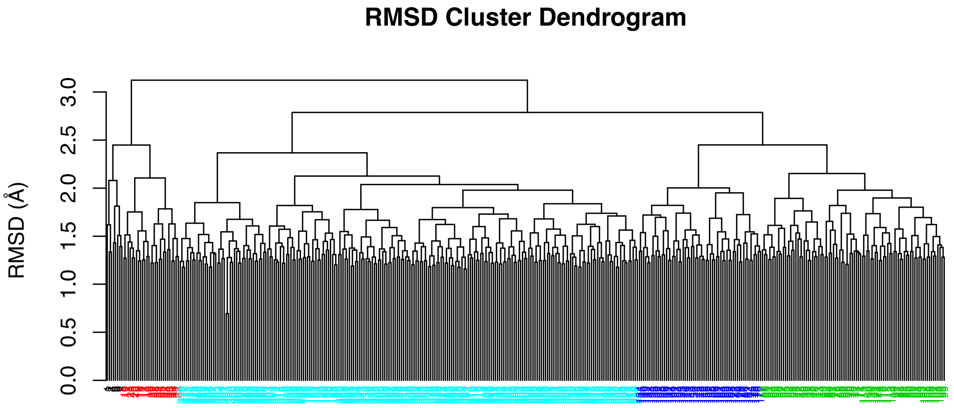


(N)

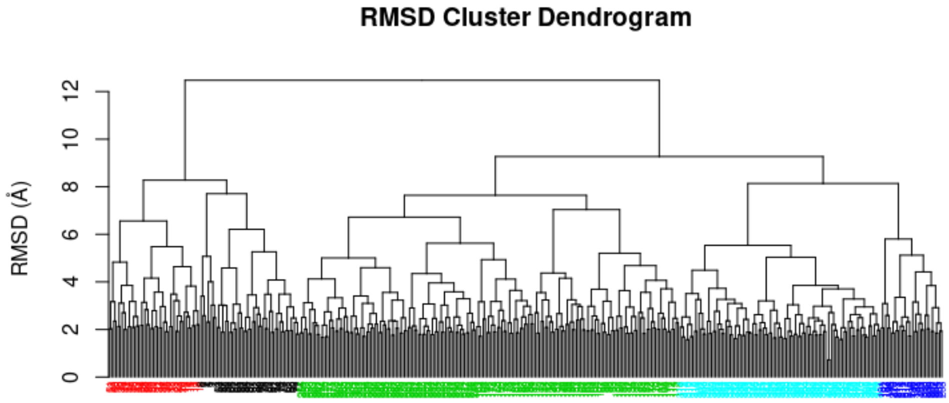


**Figure S3.** Cluster dendrogram calculated from the RMSD trajectories. Cluster dendrograms for OMF-type proteins, MFP-type proteins, and control proteins. Each color represents a cluster of frames from the MD trajectory with structural similarity. For OMF-type proteins: A) Q9I0Y7, B) Q9HXB9, C) Q9I0V8, D) Q9I006, E) Q9HU26, F) Q9HWH3, G) Q9HY88. For MFP-type proteins: H) Q9I0V5, I) Q9I0Y9, J) Q9I3R2, K) G3XD25. For control proteins L) OprM, M) MexA, N) MexB. The length of each cluster indicates the number of trajectory frames sharing structural similarity. While the image resolution may not allow for detailed viewing of cluster numbering, the relative size of each cluster reflects the population density of that conformation during the trajectory.

(A)

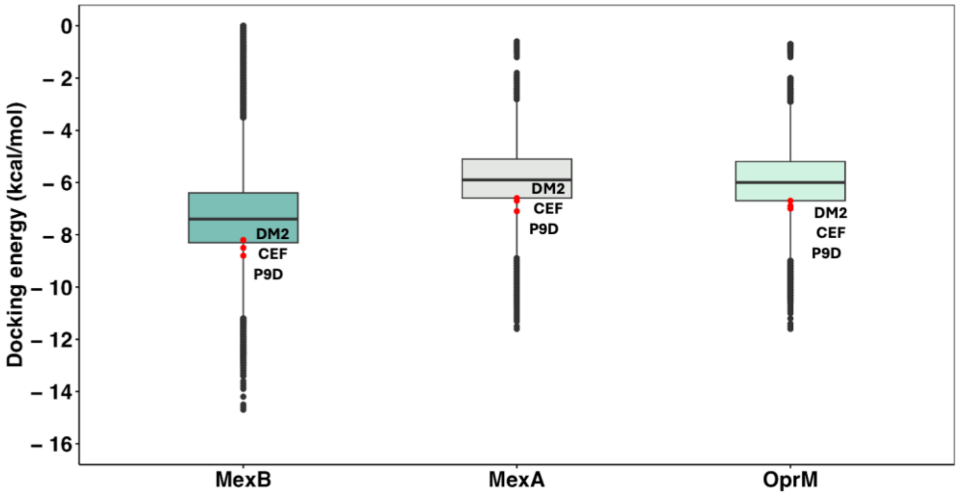


(B)

| Protein | Ligands | Q1 | Median | Q3 | Mean | S.D. |
|---|---|---|---|---|---|---|
| OprM | P9D_1 | -7.3 | -6.7 | -6.3 | -6.7 | 0.56 |
| | P9D_2 | -6.8 | -6.5 | -6.1 | -6.4 | 0.50 |
| | CEF | -7.1 | -6.8 | -6.6 | -6.9 | 0.39 |
| | DM2 | -7.2 | -6.9 | -6.7 | -7.0 | 0.53 |
| | MIY | -7.2 | -7.0 | -6.8 | -7.0 | 0.46 |
| | Oxacillin | -6.7 | -6.4 | -6.2 | -6.4 | 0.38 |
| | MBX | -7.1 | -6.7 | -6.4 | -6.7 | 0.50 |
| | Rhodamine6G | -6.5 | -6.2 | -6.4 | -6.2 | 0.39 |
| | Erythromycin | -7.3 | -7.1 | -6.7 | -7.0 | 0.64 |
| MexA | P9D_1 | -7.0 | -6.6 | -6.2 | -6.6 | 0.62 |
| | P9D_2 | -6.7 | -6.3 | -6.0 | -6.3 | 0.75 |
| | CEF | -7.0 | -6.6 | -6.3 | -6.6 | 0.45 |
| | DM2 | -7.1 | -6.7 | -6.4 | -6.8 | 0.46 |
| | MIY | -7.6 | -7.1 | -6.8 | -7.2 | 0.64 |
| | Oxacillin | -6.5 | -6.0 | -5.7 | -6.1 | 0.64 |
| | MBX | -7.1 | -6.5 | -6.1 | -6.6 | 0.71 |
| | Rhodamine6G | -6.0 | -5.8 | -5.5 | -5.8 | 0.42 |
| | Erythromycin | -7.6 | -6.9 | -6.7 | -7.1 | 0.64 |
| MexB | P9D_1 | -9.6 | -8.5 | -5.6 | -7.5 | 2.63 |
| | P9D_2 | -8.3 | -7.5 | -7.0 | -7.5 | 1.01 |
| | CEF | -8.6 | -8.1 | -6.8 | -7.7 | 1.20 |
| | DM2 | -9.1 | -8.2 | -6.6 | -7.9 | 1.51 |
| | MIY | -9.7 | -8.8 | -5.2 | -6.9 | 4.06 |
| | Oxacillin | -8.1 | -7.8 | -6.5 | -7.1 | 1.57 |
| | MBX | -8.8 | -8.0 | -7.6 | -8.1 | 0.73 |
| | Rhodamine6G | -4.6 | -3.0 | -0.3 | -1.8 | 4.55 |
| | Erythromycin | -9.5 | -8.0 | -4.1 | -6.1 | 4.32 |

**Figure S4.** Docking binding energy of drug-repurposed and control compounds. A) The distribution of docking energy for the 5,712 repurposed drugs and 8 RND pump inhibitors used as controls across the proteins MexB, MexA and OprM. The boxplots illustrate the range of docking energies, with more negative values indicating stronger binding affinities B) Descriptive statistics of the 8 control inhibitors, including the first quartile (Q1), median, third quartile (Q3), mean, and standard deviation (S.D.) of the docking energies. Note that the P9D inhibitor has two tautomer forms (P9D_1 and P9D_2), which are represented separately.

**Table S3:** Descriptive statistic of promolecular energies for the 7 OMF proteins and 4 MFP proteins. This table provides the descriptive statistics of promolecular energies for the selected drug candidates (MK-3207, R-428, and Suramin) across the studied proteins. The statistics include the first quartile (Q1), median, third quartile (Q3), mean, and standard deviation (S.D.) of the promolecular energies.

| Protein Type | Ligand | Q1 | Median | Q3 | Mean | S.D. |
|---|---|---|---|---|---|---|
| OMF | MK-3207 | -0.136 | -0.078 | -0.026 | -0.089 | 0.082 |
| | R-428 | -0.140 | -0.080 | -0.027 | -0.093 | 0.089 |
| | Suramin | -0.242 | -0.169 | -0.114 | 0.206 | 0.401 |

| | | | | | | |
|---|---|---|---|---|---|---|
| MFP | MK-3207 | -0.132 | -0.086 | -0.044 | -0.095 | 0.006 |
| | R-428 | -0.144 | -0.89 | -0.039 | -0.099 | 0.075 |
| | Suramin | -0.240 | -0.162 | -0.104 | -0.177 | 0.103 |
